# Blood-based Aβ42 increases in the earliest pre-pathological stage before decreasing with progressive amyloid pathology in preclinical models and human subjects: opening new avenues for prevention

**DOI:** 10.1007/s00401-022-02458-9

**Published:** 2022-07-07

**Authors:** Pablo Botella Lucena, Sarah Vanherle, Chritica Lodder, Manuel Gutiérrez de Ravé, Ilie-Cosmin Stancu, Ivo Lambrichts, Riet Vangheluwe, Rose Bruffaerts, Ilse Dewachter

**Affiliations:** 1 Biomedical Research Institute, BIOMED, Hasselt University, 3590 Diepenbeek, Belgium; 2 Neurology Department, ZOL Genk General Hospital, Genk, Belgium; 3 Laboratory for Cognitive Neurology, Department of Neurosciences, Leuven Brain Institute (LBI), KU, 3000 Leuven, Belgium; 4 Department of Neurology, University Hospitals, 3000 Leuven, Belgium; 5 Computational Neurology, Experimental Neurobiology Unit, Department of Biomedical Sciences, University of Antwerp, Antwerp, Belgium

## Abstract

Blood-based (BB) biomarkers for Aβ and tau can indicate pathological processes in the brain, in the early pathological, even pre-symptomatic stages in Alzheimer’s disease. However, the relation between BB biomarkers and AD-related processes in the brain in the earliest pre-pathology stage before amyloid pathology develops, and their relation with total brain concentrations of Aβ and tau, is poorly understood. This stage presents a critical window for the earliest prevention of AD. Preclinical models with well-defined temporal progression to robust amyloid and tau pathology provide a unique opportunity to study this relation and were used here to study the link between BB biomarkers with AD-related processes in pre- and pathological stages. We performed a cross-sectional study at different ages assessing the link between BB concentrations and AD-related processes in the brain. This was complemented with a longitudinal analysis and with analysis of age-related changes in a small cohort of human subjects. We found that BB-tau concentrations increased in serum, correlating with progressive development of tau pathology and with increasing tau aggregates and p-tau concentrations in brain in TauP301S mice (PS19) developing tauopathy. BB-Aβ42 concentrations in serum decreased between 4.5 and 9 months of age, correlating with the progressive development of robust amyloid pathology in APP/PS1 (5xFAD) mice, in line with previous findings. Most importantly, BB-Aβ42 concentrations significantly increased between 1.5 and 4.5 months, i.e., in the earliest pre-pathological stage, before robust amyloid pathology develops in the brain, indicating biphasic BB-Aβ42 dynamics. Furthermore, increasing BB-Aβ42 in the pre-pathological phase, strongly correlated with increasing Aβ42 concentrations in brain. Our subsequent longitudinal analysis of BB-Aβ42 in 5xFAD mice, confirmed biphasic BB-Aβ42, with an initial increase, before decreasing with progressive robust pathology. Furthermore, in human samples, BB-Aβ42 concentrations were significantly higher in old (> 60 years) compared to young (< 50 years) subjects, as well as to age-matched AD patients, further supporting age-dependent increase of Aβ42 concentrations in the earliest pre-pathological phase, before amyloid pathology. Also BB-Aβ40 concentrations were found to increase in the earliest pre-pathological phase both in preclinical models and human subjects, while subsequent significantly decreasing concentrations in the pathological phase were characteristic for BB-Aβ42. Together our data indicate that BB biomarkers reflect pathological processes in brain of preclinical models with amyloid and tau pathology, both in the pathological and pre-pathological phase. Our data indicate a biphasic pattern of BB-Aβ42 in preclinical models and a human cohort. And most importantly, we here show that BB-Aβ increased and correlated with increasing concentrations of Aβ in the brain, in the earliest pre-pathological stage in a preclinical model. Our data thereby identify a novel critical window for prevention, using BB-Aβ as marker for accumulating Aβ in the brain, in the earliest pre-pathological stage, opening new avenues for personalized early preventive strategies against AD, even before amyloid pathology develops.

**Supplementary Information:**

The online version contains supplementary material available at 10.1007/s00401-022-02458-9.

## Introduction

Brains of Alzheimer’s disease patients are characterized by amyloid plaques and neurofibrillary tangles respectively composed of aggregated Aβ and hyper-phosphorylated tau [45]. The assessment of the presence of these pathological hallmarks in combination with cognitive symptoms provides the basis for AD diagnosis [2, 6, 8, 14, 28, 44, 45, 56]. Aβ and tau-based CSF biomarkers and PET imaging can identify amyloid and tau pathology in the brain and are used for early diagnosis and even identification of subjects at risk before symptom development [2, 8, 14, 28]. Low Aβ42 concentrations, and low Aβ42/40 concentrations in CSF are validated biomarkers of amyloid pathology in brain, while high t-tau and p-tau in CSF indicate tau pathology in human subjects [4, 5, 8, 13, 14, 22, 30, 33, 44] and also in preclinical models [19, 25, 26]. Furthermore, Aβ- and tau-PET tracers enable in vivo detection of the presence of amyloid and tau pathology in humans. These biomarkers are used for diagnostic purposes for AD and experimentally even prediction of AD risk [5, 12, 34], while the scalability limits of PET or CSF analysis may hamper their use for the exponentially increasing challenge posed by AD in society.

More recently, blood-based biomarkers have emerged due to the development of ultrasensitive detection techniques encompassing mass-spectrometry-based and ultrasensitive immune assays, enabling detection of Aβ and tau in blood-based biofluids. As BB biomarkers provide an answer to the invasiveness, economical cost, accessibility and scalability issues, associated with CSF- and PET-based biomarker, they open new avenues for diagnosis, prognosis and early prevention strategies. The potential of blood-derived AD biomarkers is undeniable [3, 11, 13, 21, 22, 31, 33, 40, 47, 59], making further validation, in-depth research and understanding a high priority in the field. Studies have indicated that lower BB-Aβ42 and higher BB-Aβ40/42 or lower BB-Aβ42/40 ratio correlated with the presence of amyloid pathology in the brain of human subjects and preclinical models [13, 22, 31, 36, 37, 59]. BB-Aβ42 and BB-Aβ42/40 ratios can also be used in combination with other markers including GFAP, p-tau, or APP to indicate the presence of amyloid pathology [13, 16, 22, 31, 60]. Furthermore, BB-tau and BB-p-tau were shown to be useful biomarkers for progression to cognitive decline, for predicting progression to AD or for diagnosing AD [3, 7, 13, 15, 20–22, 26, 30, 31, 34, 38, 47, 57, 60]. Furthermore, p-Tau (p-tau 217, p-tau 181, p-tau231) in plasma and CSF have been shown to correlate with amyloid pathology [3, 4, 18, 43]. Taken together, single and combined measures of Aβ42, Aβ42/40, t-tau and p-tau are shown as useful markers for amyloid and tau positivity and predictors for progression to AD or cognitive decline in independent studies [7, 13, 15, 20, 22, 25, 26, 31, 34, 38, 40, 60].

While the relation between BB biomarkers with brain pathology, is increasingly understood, their relation with accumulation of total concentrations of Aβ and tau forms in the brain, as assessed by biochemical assays, requiring invasive analysis, is less studied. Most importantly, their relation to disease-related processes in the earliest pre-pathological phase, in absence or before development of amyloid pathology remains poorly understood. Preclinical models with well-defined temporal progression of pathology provide a unique opportunity to study this relation. As these models invariably develop amyloid pathology, the earlier preceding phase can be easily studied. In this earliest phase, Aβ and tau concentrations in the brain are anticipated to progressively increase toward reaching threshold values for aggregation. Insights in the earliest pre-pathological phase are important for personalized prevention strategies. Such strategies focus on prevention or substantial delay of amyloid pathology development by interfering with or adapting healthy lifestyle and environmental factors, by countering risk factors for AD or preventive strategies. Provided Aβ and tau-based BB biomarkers could predict accumulating concentrations of Aβ/tau in brain, efficacy of such preventive strategies could be monitored using BB biomarkers as trackers of brain-related processes. An in-depth understanding of the relation between Aβ/tau BB biomarkers and Aβ/tau-related processes in the brain, could provide a basis for understanding modifying effects of lifestyle, environmental and risk factor decreasing interventions. In this work, we aim to contribute to insights in the link between Aβ and tau-based BB biomarkers and Aβ/tau in brain, including the earliest pre-pathological phase.

In this work, we analyzed the relation between BB biomarkers and AD-related processes in the brain in preclinical models of AD, starting at the earliest pre-pathological phase, starting from 1.5 months in APPxPS1 (5xFAD) mice and 6 months of age in Tau (PS19) mice. We showed in Tau transgenic mice, increased tau concentrations in serum correlating with increased tau pathology. Increased BB tau concentrations furthermore correlated with increased p-tau and HTRF-assessed aggregated tau in the brain of tau transgenic mice. We furthermore analyzed BB-Aβ concentration, focusing in first instance on Aβ42, in view of the characteristic decrease in Aβ42 in biofluids (CSF, plasma) in the pathological phase and in view of its important role in plaque seeding. We here indeed, show an age-dependent decrease in BB-Aβ42 concentrations correlating with robust Aβ deposition and amyloid plaque pathology. But most importantly, we here show an initial increase in BB-Aβ42 concentrations, in the earliest pre-pathological stage, followed by a decrease in BB-Aβ42 when amyloid pathology was robustly present, indicating a biphasic BB-Aβ42 pattern. Increasing BB-Aβ42 in the pre-pathological stage furthermore correlated with increasing Aβ42 concentrations in the brain. Analysis in human subjects, indicated age-related changes in Aβ42 concentrations in plasma, and identified higher Aβ42 concentrations in plasma of older individuals (> 60yrs) compared to young individuals, and to AD patients. A similar increase of BB-Aβ40 concentrations was identified in the early pre-pathological phase in humans and mice, supporting the potential of the combined use of BB-Aβ42 and BB-Aβ40 at this stage. Interestingly, we here show that BB-Aβ (BB-Aβ42 and BB-Aβ40) increases in the earliest pre-pathological stage before robust amyloid plaque pathology, correlating in this phase with increasing Aβ (Aβ42 and Aβ40) concentrations in the brain. Our data may identify an interesting window for preventive strategies before amyloid pathology develops, with increasing BB-Aβ reflecting increasing Aβ concentrations in brain, validating their use as proxies. These data suggest a novel critical window for prevention, using BB-Aβ as a marker of brain-related processes, thereby opening innovative avenues for early personalized preventive strategies. These findings may not be trivial, as prevention of development of AD, may well be the most effective cure or solution for this irreversible, devastating, de-humanizing process.

## Materials and methods

### Animals

In this study, hemizygous 5xFAD mice and hemizygous TauP301S mice were used. For both strains, colonies are bred in house. The phenotype and spatio-temporal development of pathology of the in house colonies is well-characterized [32, 49, 51, 52, 62]. Hemizygous 5xFAD mice express mutant human APP695 carrying EOFAD mutations K670N/M671L (Swedish), I716V (Florida), V717I (London) and mutant human PS1 harboring 2 EOFAD mutations (M146L and L286V) driven by the thymocyte differentiation antigen 1 (ThyI) promoter, generated by the group of R. Vassar [32] (F + mice). Hemizygous TauP301S transgenic mice (PS19), express human Tau P301S (1N4R) driven by the mouse prion protein promoter (T + mice), generated by the group of V. Lee/J. Trojanowski [62]. Age- and gender-matched Tau transgenic mice (PS19) were analyzed for serum tau (*n* = 7–9) per group (n = 8, *n* = 7, *n* = 9, *n* = 9 mice respectively for 6 months, 9 months, 10.5 months and 12 months). Matching pathological and biochemical analysis was performed on these mice, and used for correlation analysis and linear regression on matched pairs. Age- and gender-matched APP/PS1 transgenic mice (5xFAD) were analyzed for serum Aβ (*n* = 10–12) per group (*n* = 10, *n* = 10, *n* = 11, *n* = 12, *n* = 11 mice respectively for 1.5 months, 3 months, 4.5 months, 6 months and 9 months). Matching pathological and biochemical analysis was performed on these 5xFAD mice with per group [*n* = 8–10 mice (*n* = 10, *n* = 10, *n* = 10, *n* = 10, *n* = 8 mice respectively for 1.5 months, 3 months, 4.5 months, 6 months and 9 months)] and used for correlation analysis and linear regression on matched pairs. For immuno-histochemical analysis, samples associated with technical issues, such as staining artifacts or damaged section in a particular brain region for immunohistochemistry, precluding correct quantification of pathology, were not included in the analysis. For biochemical analysis and biomarker analysis, values obtained out of range, precluding correct quantification were not included in the analysis. Animals were housed under regular conditions in a temperature-controlled room (20 ± 3 °C) on a 12-h day–night light cycle and with access to food and water ad libitum. All experiments were approved by the ethical committee for animal welfare of Hasselt University.

### Immunohistological analysis

Immunohistological analysis was performed as described previously [49, 51, 52]. Briefly, the brains were dissected, after 2 min transcardiac perfusion with ice-cold PBS (Sigma-Aldrich, St. Louis, USA) and fixed for 24 h in 4% PFA–PBS at 4 °C. Free-floating sagittal sections (40 μm) were generated with a vibrating HM650 V microtome (Thermo Fisher Scientific, Waltham, MA, USA) and were preserved in PBS–sodium azide 0.1%. The sagittal brain sections were first washed twice in PBS and then three times in PBS + 0.1% Triton X-100 (PBST). Permeabilization of the tissue was performed using PBST + methanol (1:1) for 10 min, and subsequently blocked with PBST + 5% milk. Free-floating sections were stained by incubation with anti-tau P-S202/T205 (AT8; Thermo Fisher Scientific, Waltham, MA, USA) and anti-Aβ (W02; Invitrogen, Carlsbad, CA, USA) antibodies. The slices were then incubated with the appropriate AlexaFluor-488 or AlexaFluor-568 coupled secondary antibodies (Invitrogen). Staining with Thiofavin S (ThioS; Sigma-Aldrich), a specific β-sheet strand intercalant, and Gallyas silver (all chemicals from Sigma-Aldrich) staining were performed on vibratome sections according to the manufacturer’s protocol as previously described [35, 49, 58, 63] and were used to demonstrate the presence of dense cored plaques and NFTs in brain sections respectively. For ThioS staining, the brain slices were mounted on 3% gelatin-coated glass slides, washed twice in PBS and incubated for 5 min in 0.3% KMnO_4_. Subsequently, the slides were washed in a solution of 1% K_2_S_2_O_5_/1% oxalic acid, followed by a solution of 1% NaBH_4_ for 20 s. Then the brain sections were incubated with 0.05% ThioS in 50% ethanol for 8 min, followed by two changes of 80% ethanol for 10 s each and three washes with large volumes of demineralized water. Slides were then placed in a high-concentrated phosphate buffer overnight in dark at 4 °C. For silver staining, the free-floating brain sections were washed in demineralized water and placed for 5 min in 5% periodic acid solution, then washed twice in demineralized water and treated for 1 min with an alkaline silver iodide solution (1 M NaOH, 0.6 M KI, 0.035% silver nitrate). Subsequently, the brain slices were washed twice for 5 min with 0.5% acetic acid solution and placed in developer solution (combining solutions A—0.5% sodium carbonate:B—0.025 M NH_4_NO_3_, 0.012 M AgNO_3_, 0.0035 M tungstosilicic acid:C—0.025 M NH_4_NO_3_, 0.012 M AgNO_3_, 0.0035 M tungstosilicic acid, 0.28% formaldehyde in a 10:3:7 ratio) for 5 min. Then the brain slices were rinsed twice in 0.5% acetic acid, washed with demineralized water and placed in 0.1% gold chloride solution for 5 min followed by a 1% sodium thiosulphate solution for 5 min and a final wash in water. All chemicals used were from Sigma-Aldrich. Images were acquired with a Leica DM400 B LED fluorescence microscope (Leica, Diegem, Belgium), silver staining was assessed using a bright field microscope. All images were analyzed using ImageJ open-source software (National Institutes of Health, Bethesda, MD, USA). Quantitative analysis of tau pathology was performed on AT8 stained vibratome sections. Well-defined sagittal sections at 1.32 mm lateral from bregma were selected for quantification of AT8 positive pathology. Tau pathology was analyzed by measuring the area occupied by tau tangles relative to the total image area of the brain regions of interest, using Image J software (U.S. National Institutes of Health, Bethesda, MD, USA). Amyloid pathology was analyzed in a similar way by measuring the W02 positive area and ThioS area, respectively. The extent of amyloid pathology and tau pathology in the different brain regions was analyzed by measuring the positively stained W02, AT8 area % relative to the total brain region area. Surface area were measured by delineating the brain structures as defined by the Mouse brain atlas and measuring the structure’s absolute surface area in µm2 on 5 × digital images using Image J software.

### Blood collection mice

Blood was obtained during dissection by cardiac puncture. Samples were collected in polypropylene tubes and stored at room temperature for 15 min and subsequently centrifuged for 10 min at 2500*g*. The supernatants were stored at − 28 °C until further analysis. Prior to dissection, mice were anesthetized intraperitoneally using a mixture of ketamine/xylazine (Nimatek/Rompun). For in vivo sampling, serum was collected by needle puncture of the facial vein. For the longitudinal analysis blood samples were collected triweekly, by needle puncture of the facial vein, from 2.5 months onwards, at 11 sequential time points. The blood samples were collected in polypropylene tubes stored at room temperature for 15 min, and then centrifuged at 2500*g* for 10 min. Following centrifugation, supernatant (serum) was collected in polypropylene tubes and stored at − 28 °C until further use.

### Biochemical analysis

Tau aggregates concentrations in total brain homogenates were measured using the homogeneous time resolved fluorescence (HTRF) tau aggregation kit (6FTAUPEG; Cisbio, Perkin-Elmer, FR) according to previously validated protocols in our group, following the manufacturer protocol [50]. After incubation overnight of total brain homogenates of Tau transgenic mice with the anti-tau-d2 antibody and anti-tau-tb antibody at room temperature on a 384-well plate (Cisbio), the HTRF signals were measured with a TECAN Safire 2 microplate reader (Tecan Group Ltd., CH). The levels of aggregated tau (Delta F%) were calculated using the ratios of the two emission signals (620 and 665 nm), according to the manufacturer formula. HTRF-tau aggregates values were previously shown to correlate with tau pathology in our tau model [50]. Measurements of Aβ42 and Aβ40 concentrations in brain were measured using the MSD VPLEX Plus Aβ Peptide Panel 1 (6E10) Kit (K15200G-1). Measurements of t-tau and p-tau concentrations in brain homogenates were measured using the Phospho(Thr231)/Total Tau kit (K15121D). Mouse serum levels of Aβ and tau were determined on the mesoscale discovery (MSD) platform Quickplex SQ 120 (Rockville, MD). Mouse Aβ42 and Aβ40 concentrations in serum were measured using the MSD VPLEX Plus Aβ Peptide Panel 1 (6E10) Kit (K15200G-1). Measurements of concentrations were performed based on a standard dilution curve including 4 samples between 0 and 7187 pg/ml. The concentrations of t-tau and p-tau in mice serum were measured using the Phospho(Thr231)/Total Tau kit (K15121D). Aβ42, Aβ40, t-tau, and p-tau were measured in biofluids of human subjects. Human CSF concentrations of Aβ42, Aβ40, p-tau-thr181, and t-tau measured using commercially available ELISAs (INNOTEST^®^ β-Amyloid _(1–42),(1–40)_, Phospho-Tau_(181-p)_, hTau Ag (Fujirebio Europe N.V., Ghent, Belgium) according to the manufacturer’s recommendations. Plasma levels of Aβ40 and Aβ42 were measured simultaneously using the commercially available Simoa Human Neurology 3-Plex Assay Kit on the automated SR-X-analyzer (Quanterix, Lexington, MA, USA).

### Tissue homogenization

Brains were dissected following anesthesia and trans-cardiac perfusion with ice-cold saline, tissue samples were snap-frozen in liquid N_2_ and stored at − 80 °C. Cortices were homogenized using a Potter-Elvejhem homogenizer (20 strokes, 700 rpm; VWR, Leuven, Belgium) in 6 vol of ice-cold Tris-proteinase-phosphatase-inhibitor buffer, containing 25 mM Tris–HCl (pH 8.1), 150 mM sodium chloride (Sigma), 1 mM ethylene diamine tetra-acetic acid (EDTA, Merck), 1 mM ethylene glycol tetra-acetic acid (EGTA, Sigma-Aldrich), 5 mM sodium pyrophosphate (Sigma), 5 mM sodium fluoride (Sigma-Aldrich), 1 mM PMSF (Sigma), 1 mM sodium vanadate (Sigma-Aldrich), and a cocktail of proteinase inhibitors (Roche) and phosphatase inhibitors (Roche).

### Study participants and biofluid collection

The study was initiated following approval by the ethics committees of Ziekenhuis Oost Limburg (ZOL) and Hasselt University and executed in accordance with the Good Clinical practice Guidelines. Human subjects or when applicable their caretaker provided written consent before enrollment in the study. Study participants were recruited from the neurology department of Ziekenhuis Oost Limburg. All participants underwent standard clinical examination and neuropsychological testing. A clinical diagnosis by trained neurologists and was assigned at consensus diagnostic meetings. Diagnosis of biomarker proven AD was made based on the standard criteria NINCDS-ADRDA. The control groups comprised non-AD controls. Final diagnoses in this group included suspected meningitis, ALS, MS, progressive supra-nuclear palsy. After written consent, lumbar punctures were performed at the neurology department of ZOL. CSF was immediately aliquoted into polypropylene storage tubes, immediately transferred and stored at − 80 °C before use. Blood samples were collected at the same time. Blood was collected via venepuncture. To obtain plasma, blood was collected into tubes containing EDTA anti-coagulants (6 ml BD Vacutainer K2EDTA tube, BD Diagnostics). Plasma samples were centrifuged (1500*g*, 10 min), collected and aliquoted in polypropylene tubes, and stored at − 80 °C in the University Biobank of Limburg (UBiLim). Demographic and clinical characteristics of all study participants are detailed in Supplementary Table 1.

### Statistical analysis

**Fig. 1:**
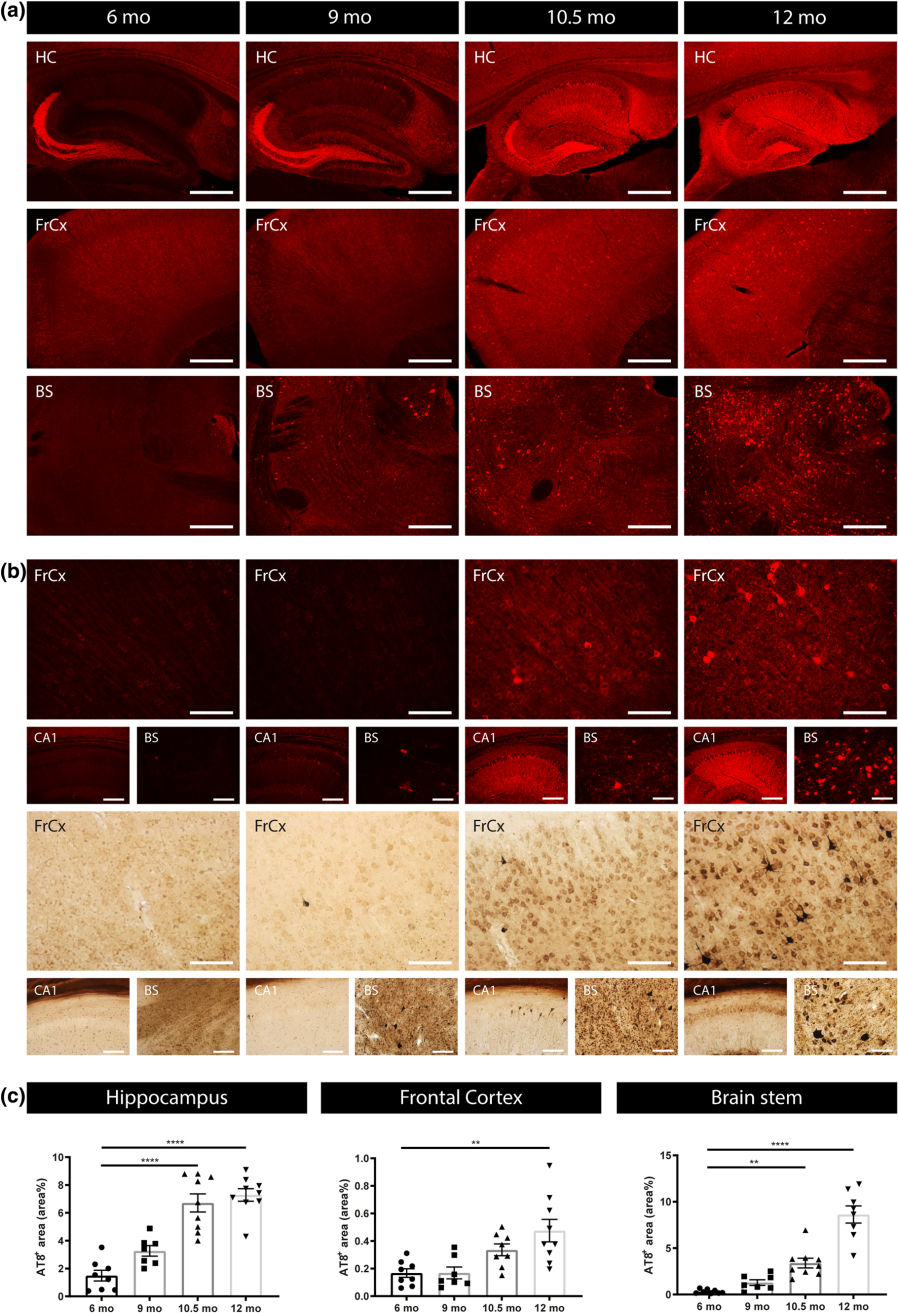
Progressive tau pathology in Tau P301S mice.** a** Representative images of anti-p-tau (pSer202/Thr205; AT8) staining in Hippocampus (HC), Frontal Cortex (FrCx) and Brain Stem (BS) region of Tau mice (Tau^P301S^/TPS) at 6 months, 9 months, 10.5 months and 12 months are presented. AT8 staining is significantly increased at 10.5 and 12 months of age compared to 6 months of age. **b** Higher magnifications of representative images showing AT8 staining (upper row) and Gallyas silver staining (lower row), indicating the presence of neurofibrillary tangles. **c** Quantitative analysis of AT8 staining in hippocampus, frontal cortex and brain stem (from left to right) of 6-month-, 9-month-, 10.5-month- and 12-month-old Tau mice. One‐way ANOVA with Tukey’s multiple comparison test (normally distributed); Kruskal–Wallis test with Dunn’s multiple comparison (non‐normally distributed). (6 months: *n* = 8; 9 months: *n* = 7; 10.5, 12 months: *n* = 9). Data are presented as means ± SEM; *p < 0.05; **p < 0.01; ***p < 0.001; ****p < 0.0001. *HC* hippocampus, *FrCx* frontal cortex, *BS* brain stem

Data were statistically analyzed using GraphPad Prism version 9.0 (GraphPad Software Inc, San Diego, USA). Normal distribution was tested using Shapiro–Wilk test. Data were analyzed using one-way analysis of variance (ANOVA) with Dunnett’s test for multiple comparison for normally distributed data, or Kruskal–Wallis test with Dunn’s multiple comparison test for non-normally distributed data. Unpaired *t* test was used for data presented in FigS1. Repeated measures one-way ANOVA with Dunnett’s multiple comparison test was used for the longitudinal analysis. For this analysis missing values, due to lack of sample or due to technical errors were filled in using mean of values method. Correlation analysis was performed using linear regression analysis, Spearman’s correlation analysis and Pearson’s correlation analysis, as appropriate. In all analyses, missing values present technical errors including damaged section or artifacts precluding quantification of immunohistochemistry, values outside range for biochemical analyses (ELISA, MSD, SIMOA, HTRF) or lack of sample due to technical issue with sample collection. Outliers were identified using ROUT 0.1%. Results were presented as mean ± standard error (SEM). A probability of p < 0.05 was considered significant. *p < 0.05, **p < 0.01, ***p < 0.001, ****p < 0.0001.

## Results

### Serum tau concentrations increase with development of tau pathology in brain in a preclinical tauopathy model

**Fig. 2:**
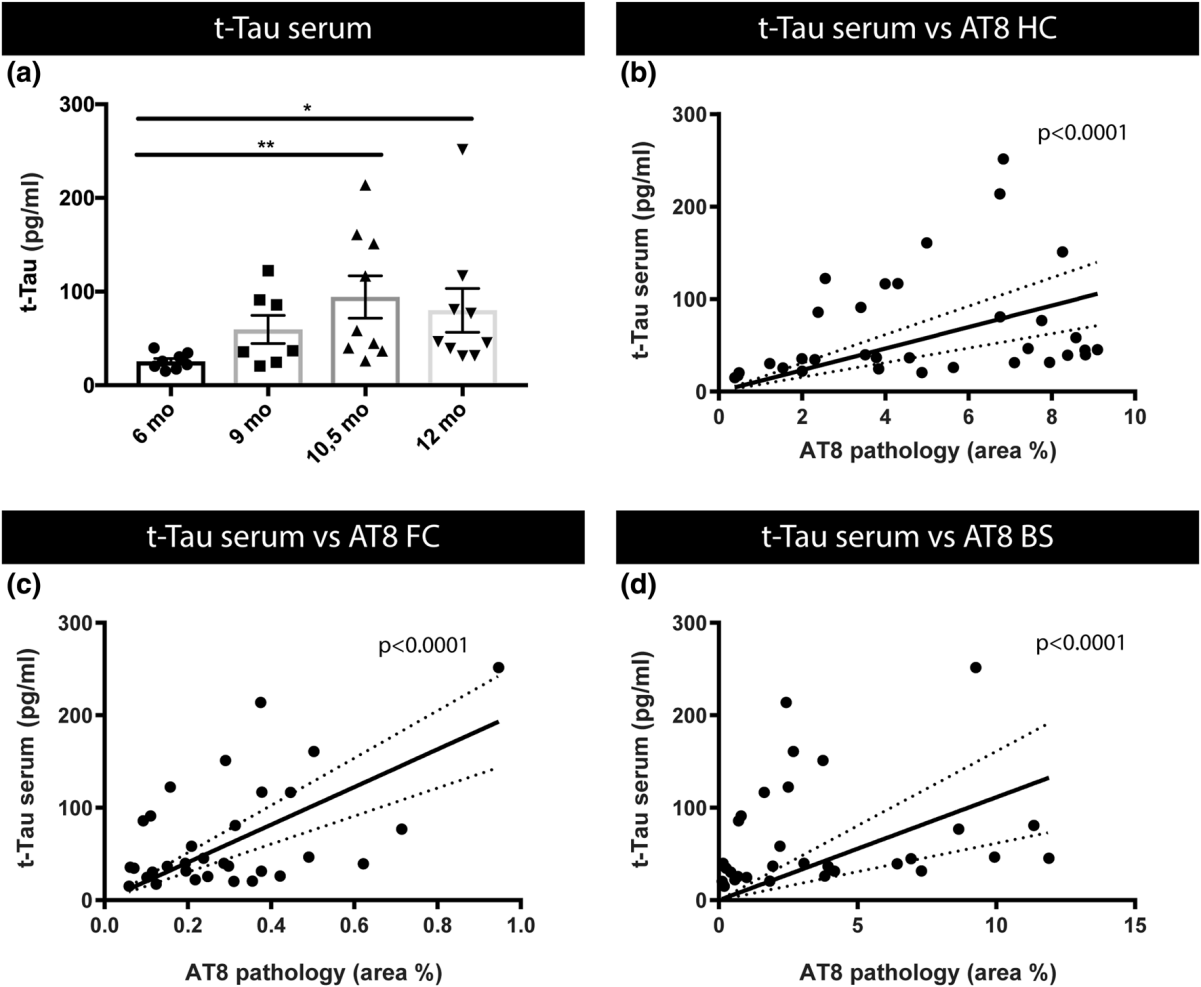
Tau serum concentrations in relation to tau pathology in Tau P301S mice.** a** Tau concentrations in serum (t-tau) of 6-month-, 9-month-, 10.5-month- and 12-month-old Tau mice were measured using electrochemiluminescence assay, revealing an age-dependent increase, being significantly increased at 10.5 and 12 months compared to 6 months of age. Kruskal–Wallis test with Dunn’s multiple comparison (6 months: *n* = 8; 9 months: *n* = 7; 10.5, 12 months: *n* = 9). Data are presented as means ± SEM; *p < 0.05; **p < 0.01; ***p < 0.001; ****p < 0.0001. **b**–**d** Spearman’s correlation between t-tau serum concentrations and AT8 staining in HC (*r*_s_ = 0.4549, *p* < 0.01, *n* = 33), FrCx (*r*_s_ = 0.3919, *p* < 0.05, *n* = 32), and BS (*r*_s_ = 0.482, *p* < 0.01, *n* = 32). Linear regression analysis furthermore demonstrated that the slope was significantly different from zero for t-tau serum levels in relation to tau pathology in HC (*p* < 0.0001) (**b**), FrCx (*p* < 0.0001) (**c**) and BS (*p* < 0.0001) (**d**)

To study the relation between blood-based biomarkers dynamics and tau pathology in the brain of a preclinical tauopathy model, we performed a cross-sectional analysis in TauP301S transgenic mice at different ages. TauP301S mice (PS19 mice) develop a robust and reproducible neurodegenerative phenotype [24, 49, 51, 52, 62]. This encompasses the development of tau pathology in brain stem, cortex and hippocampus, in combination with hippocampal and cortical atrophy (Fig. 1), and the development of progressive motor deficits, followed by premature death [24, 49, 51, 52, 62] (Supplementary Fig. S1, online resource). This phenotype is consistently displayed in independent research groups, indicating TauP301S mice as a robust model to analyze the relation between BB biomarkers and pathological processes in the brain, in the pathological as well as the earliest pre-pathological stage. In the TauP301S cohort routinely used in our lab, this phenotype starts at the age of ± 10–11 months (Fig. 1 and Supplementary Fig. S1, online resource). Staining of tau pathology was performed using immunostaining with AT8, at 6 months, 9, 10.5 and 12 months of age. For staining tau pathology, we here used an AT8 staining protocol, previously optimized for staining tau pathology strongly correlating with aggregated tau, further demonstrated by silver staining of a strongly comparable pattern as observed with AT8 staining (Fig. 1). AT8 staining demonstrated the progressive development of tau pathology in TauP301S mice. Tau pathology is absent at 6 months, while only very scarcely, neurons with tau pathology are detected at the age of 9 months. However, from 10.5 months onwards, tau pathology is clearly present and further aggravated at the age of 12 months. Quantitative analysis revealed a significant increased tau pathology in mice of 10.5 and 12 months old compared to 6-month-old TauP301S mice (Fig. 1). To assess the relation between tau serum concentrations and pathological processes in the brain, we collected blood samples of the cross-sectional cohort of mice. Serum total tau concentrations were measured using sensitive electrochemiluminescence detecting total tau. This demonstrated a significant increase in serum total tau concentrations in mice of 10.5 and 12 months old compared to 6-month-old TauP301S mice. A strikingly similar significant difference in tau pathology in different brain regions was found in the 10.5- and 12-month-old age group compared to the 6 months’ age group. We performed Spearman’s correlation analysis, demonstrating a significant correlation between t tau serum concentrations and tau pathology in cortex (*p* value < 0.05; *r*_s_ 0.39), hippocampus (*p *value < 0.01; *r*_s_ 0.45) and brain stem (*p* value < 0.01; *r*_s_ 0.48) in Tau P301S mice (Fig. 2). Linear regression analysis furthermore demonstrated that the slope was significantly different from zero for t-tau-serum levels in relation to tau pathology in hippocampus (*p *value < 0.0001), brain stem (*p *value < 0.0001) and cortex (*p *value < 0.0001) (Fig. 2). Using this assay, we also measured p-tau-Thr231 in serum in Tau mice. Serum p-tau-Thr231 was significantly increased in the 10.5-month-old group compared to the young 6-month-old group, relating with development of tau pathology. Linear regression analysis demonstrated a slope significantly different from 0 between serum p-tau-Thr231 and tau pathology in hippocampus (*p*-value < 0.0001), frontal cortex (p-value < 0.0001) and brain stem (*p*-value < 0.01) (Supplementary Fig. S2, online resource), in line with findings in human cohorts [3]. Taken together, our data analyzing BB-t-tau concentrations are in line with previous findings in preclinical models and in human subjects, showing a correlation between tau-serum concentrations and the presence of tau pathology in the brain.

### Serum total tau concentrations correlate with p-tau and aggregated tau in brain homogenates of a preclinical tauopathy model

**Fig. 3:**
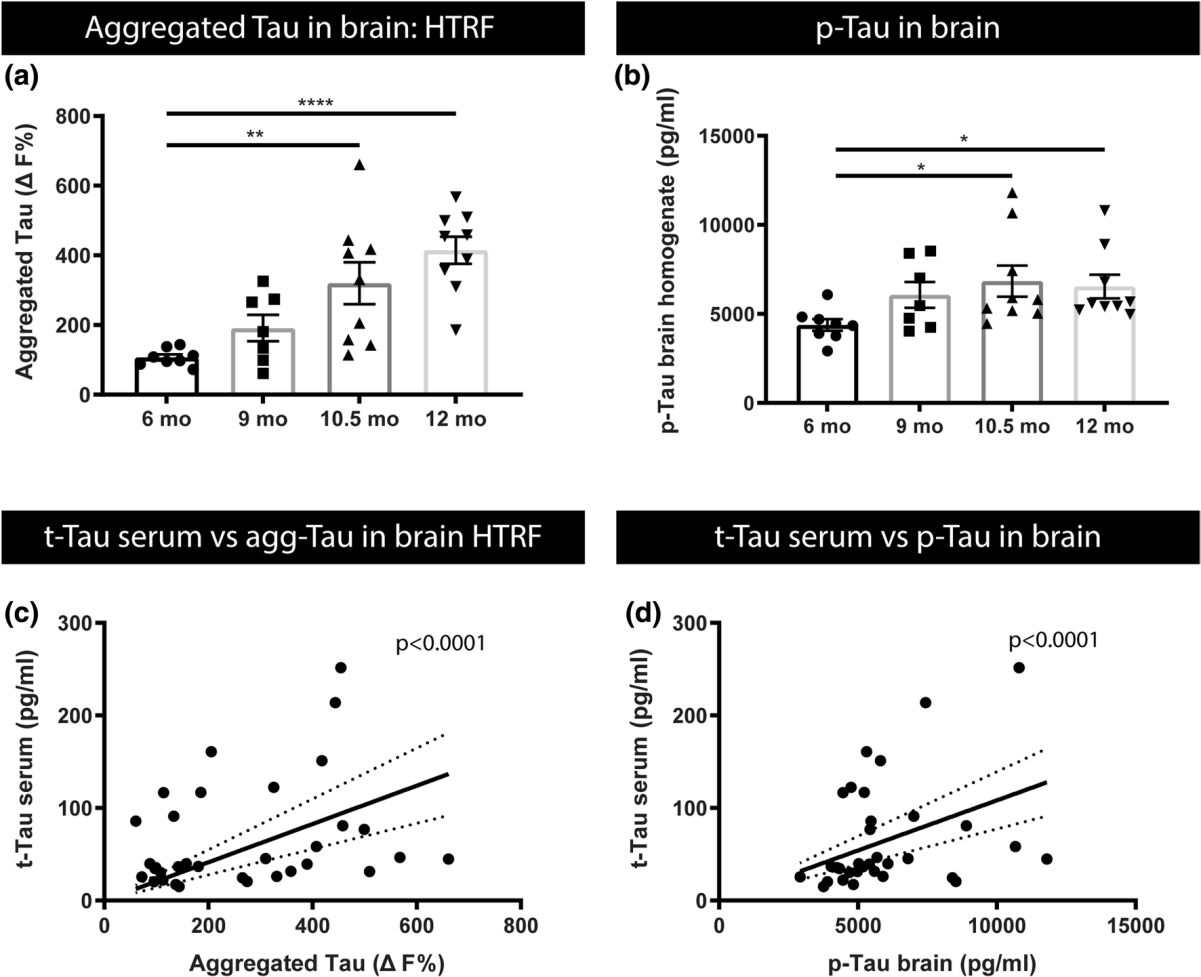
Relation between t-tau in serum and concentrations of p-tau and tau aggregates in brain in Tau P301S mice.** a** Concentrations of tau aggregates in brain extracts of 6-month-, 9-month-, 10.5-month- and 12-month-old Tau mice were quantified using homogeneous time resolved fluorescence (HTRF) assay, revealing an age-dependent increase, being significantly increased at 10.5 and 12 months compared to 6 months. One‐way ANOVA with Tukey’s multiple comparison test (normally distributed) Data are presented as mean ± SEM, *p < 0.05; **p < 0.01; ***p < 0.001; ****p < 0.0001; (6 months: *n* = 8; 9 months: *n* = 7; 10.5, 12 months: *n* = 9). **b** Quantitative analysis of p-tau-Thr231 in brain extracts of 6-month-, 9-month-, 10.5-month- and 12-month-old Tau mice, measured by MSD p-tau-Thr231 assay, reveals an age-dependent increase in p-tau-Thr231 in the brain at 10.5 and 12 months of age compared to 6-month-old mice. Kruskal–Wallis test with Dunn’s multiple comparison (non‐normally distributed). Data are presented as mean ± SEM; *p < 0.05; **p < 0.01; ***p < 0.001; ****p < 0.0001. (6 months: *n* = 8; 9 months: *n* = 7; 10.5, 12 months: *n* = 9). **c**, **d** Spearman’s Correlation between t-tau serum concentrations and concentrations of aggregated tau in brain (*r*_s_ = 0.362, *p* < 0.05, *n* = 33) and, p-tau in brain (*r*_s_ = 0.4212, *p* < 0.05, *n* = 33). Linear regression analysis furthermore demonstrated that the slope was significantly different from zero for tau serum levels in relation to aggregated tau (HTRF, *p* < 0.0001) (**c**) and p-tau (*p* < 0.0001) (**d**) in brain homogenates

The relation between tau-serum concentrations and pathological processes in the brain in a tauopathy model, was further assessed by studying the relation between serum tau concentrations and concentrations of phosphorylated tau and aggregated tau in brain homogenates of TauP301S mice. We measured concentrations of p-tau-Thr231 in total brain homogenates of Tau P301S mice at 6, 9, 10.5 and 12 months of age using a p-tau-Thr231 electrochemiluminescence assay. This demonstrated significantly increased concentrations of p-tau-Thr231 at 10.5 and 12 months of age compared to 6-month-old TauP301S mice (Fig. 3). To further assess age-dependent tau alterations in brain of TauP301S mice, we used a homogeneous time resolved fluorescence (HTRF)-based assay that measures aggregated tau, ranging from small to large tau oligomers and aggregates. In this assay, tau aggregates are measured using a sandwich immunoassay with an anti-tau monoclonal antibody as donor and acceptor. When donor and acceptor are in close proximity, the excitation of the HTRF donor will lead to an energy transfer (FRET) to the HTRF acceptor, generating a specific HTRF signal. This HTRF signal is proportional to the amount of tau aggregates (Fig. 3), including smaller and larger tau aggregates. We measured aggregated tau in total brain homogenates of TauP301S mice at 6, 9, 10.5, and 12 months of age. This demonstrated significantly increased aggregated tau concentrations in 10.5- and 12-month-old tau mice compared to 6-month-old mice (Fig. 3). These findings relate to significantly increased serum t-tau concentrations at 10.5 and 12 months of age compared to 6-month-old TauP301S mice (Fig. 3c, d). Spearman’s correlation analysis demonstrated a significant correlation between t-tau serum concentrations and aggregated tau (HTRF) as well as p-tau-Thr231 in total brain homogenates of TauP301S mice (*p *value < 0.05; *r*_s_ 0.36 and *p *value < 0.05; *r*_s_ 0.42). Linear regression analysis demonstrated a highly significant linear relation (slope significantly different from 0 with *p*-value < 0.0001) between t-tau serum concentrations and p-tau concentrations and aggregated tau concentrations in total brain homogenates (Fig. 3c, d). Linear regression furthermore demonstrated significant relation between serum p-tau-Thr231 and p-tau-Thr231 in brain (*p*-value < 0.0001) and HTRF-assessed tau aggregates (*p*-value < 0.0001) (Supplementary Fig. S2, online resource). Taken together, increases in serum tau concentrations reflect ongoing pathological processes in the brain, correlating with tau aggregation, pathology and tau-phosphorylation in this preclinical model of tauopathy.

### Age-dependent changes in Aβ concentrations in serum in a preclinical model of amyloid pathology reveals a biphasic pattern of Aβ42

**Fig. 4:**
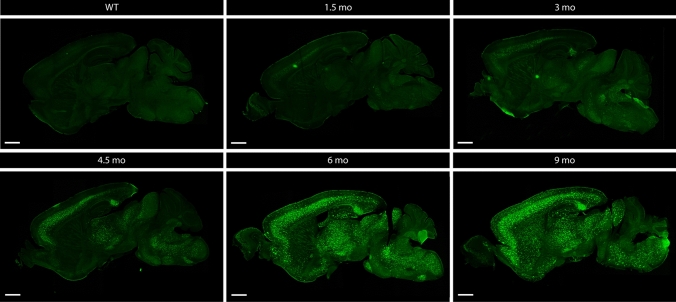
Full brain overview of the progression of amyloid pathology in 5xFAD mice. Representative overview images of full brain sections of 5xFAD mice following WO2 staining at 1.5 months, 3 months, 4.5 months, 6 months and 9 months of age and a wildtype mouse at 9 months of age, showing progression of amyloid pathology in 5xFAD mice throughout the brain

**Fig. 5:**
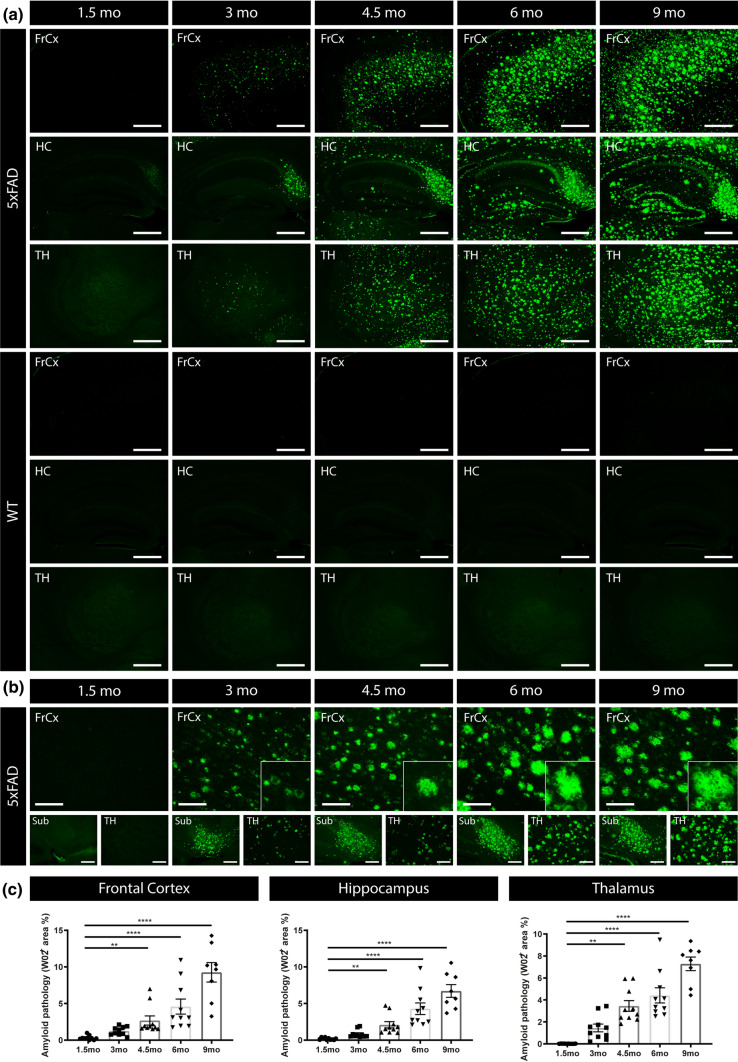
Progressive amyloid pathology in 5xFAD mice.** a **Representative images of anti‐Aβ (W02) stainings of 1.5-month-, 3-month-, 4.5-month-, 6-month- and 9-month-old 5xFAD mice in Frontal Cortex (FrCx), Hippocampus (HC), and Thalamus (TH), show an age-dependent increased deposition of amyloid plaques. Representative images of age-matched wild type mice are presented for comparison. **b** Higher magnifications of representative images of WO2 staining in 5xFAD mice in Frontal Cortex in the different age groups are presented. **c** Quantitative analysis of WO2 staining in 5xFAD mice of 1.5 months, 3 months, 4.5 months, 6 months and 9 months of age reveals a significant increased amyloid pathology from 4.5 months of age onwards compared to the 1.5 months age group in FrCx, HC and TH. Kruskal–Wallis test with Dunn’s multiple comparison (non‐normally distributed). Data are presented as mean ± SEM; *p < 0.05; **p < 0.01; ***p < 0.001; ****p < 0.0001. *FrCx* frontal cortex, *HC* hippocampus, *TH* Thalamus) (1.5 months, 3 months, 4.5 months, 6 months and 9 months: *n* = 10, 10, 10, 10, 8; for all groups: *n* = 8–10)

**Fig. 6:**
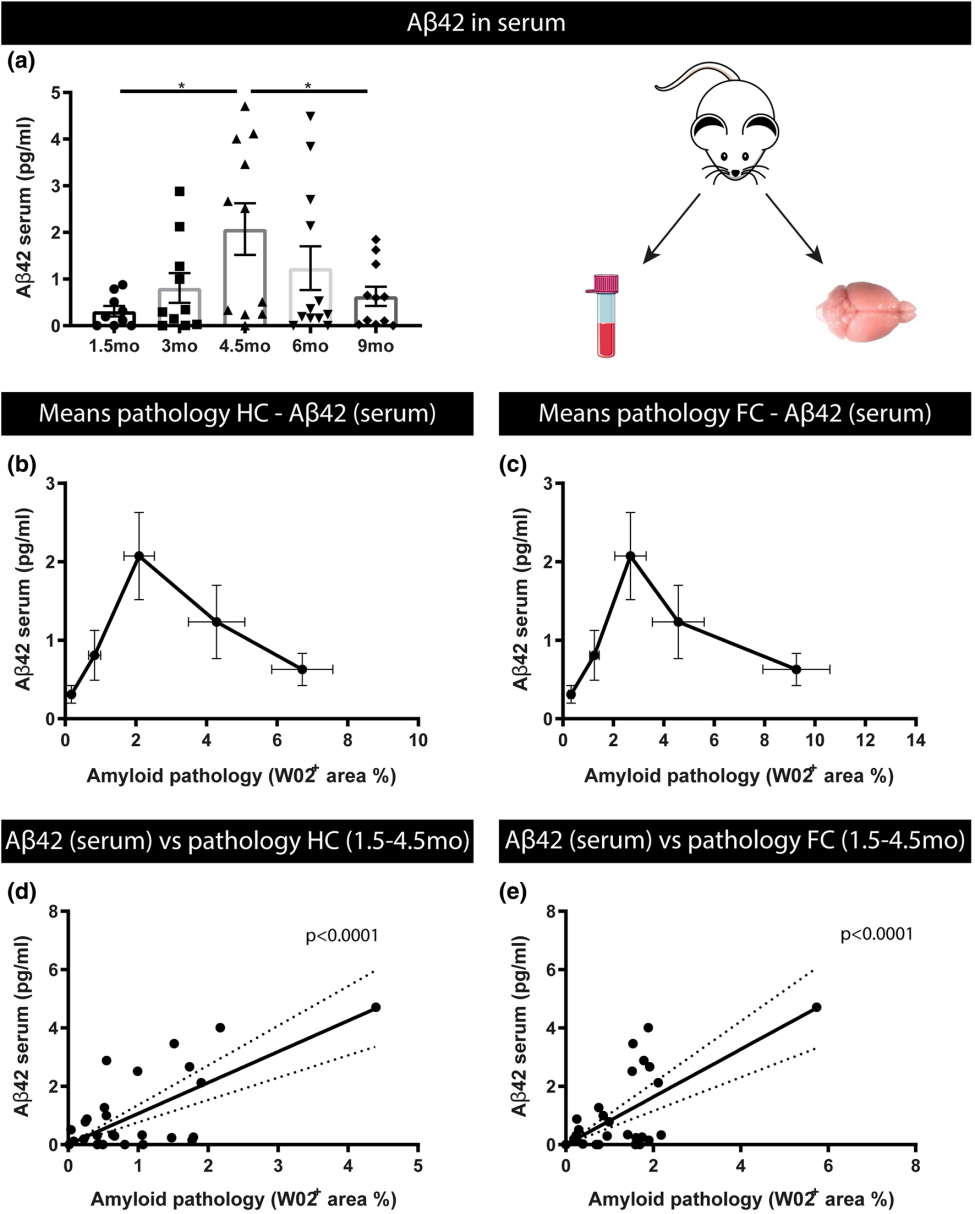
Relation between serum Aβ42 concentrations and amyloid pathology in different brain regions in 5xFAD mice.** a** Aβ42 concentrations measured in serum of 1.5-month-, 3-month-, 4.5-month-, 6-month- and 9-month-old 5xFAD mice. Quantitative analysis reveals significantly higher concentrations in serum of 4.5 months 5xFAD mice compared to 1.5-month-old, and compared to 9-month-old mice. This reveals a biphasic pattern with an initial rise in the concentration of Aβ42 at 4.5 months, preceding a decrease in the Aβ42 levels. One‐way ANOVA with Tukey’s multiple comparison test (normally distributed) Data are presented as mean ± SEM; *p < 0.05; **p < 0.01; ***p < 0.001; ****p < 0.0001 (1.5, 3, 4.5, 6 months, 9 months: *n* = 9, 10, 11, 12, 11, for all groups *n* = 9–12). **b**, **c** Dynamics of Aβ42 serum levels and pathology mean (WO2 positive area %) in HC (**b**) and FC (**c**) per age group. **d**, **e** Spearman´s correlation analysis between Aβ42 serum levels and pathology (WO2 positive area %) in HC (*r*_s_ = 0.46, *p* < 0.05, *n* = 29) and FrCx (*r*_s_ = 0.51, *p* < 0.01, *n* = 29) between 1.5 and 4.5 months of age. Linear regression analysis furthermore demonstrated that the slope was significantly different from zero for Aβ42 serum levels and pathology (WO2 positive area %) in HC (*p* < 0.0001) (**d**) and FrCx (p < 0.0001) (**e**)

We next assessed the relation between Aβ concentrations in serum with Aβ-related processes in the brain and development of amyloid pathology, using 5xFAD mice, a well-characterized preclinical model of amyloid pathology. Hereto, we performed a detailed cross-sectional analysis in 5xFAD transgenic mice at different ages. 5xFAD transgenic mice reproducibly and consistently develop early amyloid pathology and behavioral deficits, including cognitive dysfunction, as demonstrated by independent research groups [32, 51]. In our cohort routinely used in the lab, amyloid plaques start to develop at the age of 3–4 months, starting with the formation very scarce and small amyloid plaques in subiculum and followed later by more widespread pathology in cortex [32, 51]. Staining of amyloid pathology was performed using immunostaining with WO2, to assess total plaque amyloid load. Pathological analysis was performed at the age of 1.5, 3, 4.5, 6 and 9 months of age. Amyloid pathology was absent at 1.5 months of age (Figs. 4, 5). Mostly intra-neuronal and scarce small amyloid deposits of Aβ were detected at 3 months of age. While amyloid plaques were significantly detected at 4.5 months of age in hippocampus (HC), frontal cortex (FC) and thalamus (TH). Quantitative analysis revealed significant amyloid pathology starting at the age 4.5 months of age and all older age groups, i.e., 6 and 9 months of age, compared to mice of 1.5-month-old (Fig. 5c). ThioS staining was performed to assess the load of compacted amyloid plaque cores, showing similar results, i.e., significant amyloid pathology in 4.5-, 6- and 9-month-old mice compared to 1.5-month-old mice (Supplementary Fig. S3, online resource). To study the potential of blood-based Aβ (Aβ42 and Aβ40) as biomarker for brain-related changes, we collected serum samples in our cross-sectional cohort of 1.5-, 3-, 4.5-, 6- and 9-month-old 5xFAD mice. In view of characteristic decreasing Aβ42 concentrations in biofluids associated with amyloid pathology, we first analyzed Aβ42 concentrations, using an electrochemiluminescence assay detecting different Aβ forms. We quantified Aβ42 in serum of 5xFAD mice of the different age groups. This revealed a significant decrease in Aβ42 concentration between 4.5 months of age and 9 months of age, correlating with the development of significant and robust amyloid pathology (Fig. 6a), correlating with progressive development of widespread and large plaques. These findings are in line with previous data demonstrating decreased Aβ42 concentrations in blood-based biofluids, correlating with increased amyloid pathology in human subjects [11, 13, 16, 19, 22, 31, 33, 36, 40, 59]. Interestingly, Aβ42 concentrations were significantly increased in serum at 4.5 months of age compared to 1.5-month-old 5xFAD mice (Fig. 6a). This phase corresponds with the earliest pre-pathological phase, before development of robust amyloid pathology (Fig. 5). This finding indicates significantly increased Aβ42 serum concentrations at a time point when Aβ concentrations are anticipated to increase in the brain to reach a threshold value for aggregation. This phase may present an ideal —even critical— time window for preventive strategies. Prevention of the build-up of Aβ42 concentrations in this early phase before reaching threshold levels of Aβ42, may significant postpone or effectively prevent amyloid plaque formation.

Using this electrochemiluminescence assay, we also measured Aβ40 concentrations. Similar results were found for Aβ40 concentrations in serum, in the earliest pre-pathological phase between 1.5 and 4.5 months, showing increasing BB-Aβ40 (Supplementary Fig. S4, online resource). No significant decrease for BB-Aβ40 was measured in the pathological phase between 4.5 and 9 months. Also no significant changes were identified in the BB-Aβ42/Aβ40 ratio in the earliest pre-pathological phase, while BB-Aβ42/Aβ40 ratio significantly decreased in the pathological phase (Supplementary Fig. S5, online resource). These findings are in line with decreased BB-Aβ42 and decreased BB-Aβ42/Aβ40 ratio in the pathological stage in human cohorts. It must be noted that in our cohort of 5xFAD mice, measurements of serum p-tau-thr231 and serum t-tau, did not yield values above threshold, precluding their analysis in 5xFAD mice, and the relation of BB-tau with amyloid pathology in our model. Different experimental setups will be required for BB-p-tau and BB-t-tau analysis, extending beyond the current study focusing on the relation with BB-Aβ. Most importantly, BB-Aβ increased in the early pre-pathological stage.

We next assessed the relation of BB-Aβ with developing amyloid pathology in 5xFAD mice. Quantitative analysis of amyloid load in hippocampus and frontal cortex, in combination with serum Aβ42 concentrations per age group, revealed a biphasic relation with serum Aβ42 concentrations initially increasing to reach maximal concentrations in the 4.5 age group (Fig. 6b, c) subsequently followed by decreasing serum Aβ42 concentrations (Fig. 6b, c). To further study the relation between serum Aβ42 concentrations and development of amyloid pathology, we performed correlation analysis using a biphasic paradigm taking 4.5 months as a turning point. We performed correlation analysis on the data collected between 1.5 and 4.5 months, and from 4.5 until 9 months of age (Fig. 6d, e). Spearman’s correlation analysis and linear regression analysis revealed a significant positive correlation between Aβ42 concentrations in serum and increasing amyloid deposition in the brain in the earliest phase (1.5 until 4.5 months of age) of the Aβ aggregation process (Spearman’s correlation analysis (FC: *p*-value < 0.01; *r*_s_ 0.51 and HC: *p*-value < 0.05; *r*_s_ 0.46)) (Fig. 6). Also for Aβ40 linear regression and Spearman’s correlation revealed a significant positive correlation between Aβ40 concentrations and pathology between 1.5 and 4.5 months, in this early phase (FC (*r*_s_ = 0.464, *p* < 0.05) and HC (*r*_s_ = 0.4759, p < 0.05) (Supplementary Fig. S4, online resource). In the later stage, with progressive amyloid pathology development, from 4.5 months until 9 months of age, a negative correlation between Aβ42 serum concentrations and amyloid pathology was demonstrated (Supplementary Fig. S6, online resource). The latter is in line with previous data in preclinical models and human subjects.

Taken together, a biphasic dynamic of Aβ42 was observed, with a significant positive correlation in the earliest pre-pathological phase, before progressive development of robust amyloid pathology. Most importantly, we here show increased BB-Aβ (BB-Aβ42 and BB-Aβ40) concentrations in serum in the earliest pre-pathological stage, followed by decreasing BB-Aβ42 and ratio of BB-Aβ42/40 in the pathological stage.

### Serum Aβ concentrations correlate with increasing Aβ concentrations in brain homogenates in the earliest pre-pathological phase in a preclinical model of amyloid pathology

**Fig. 7:**
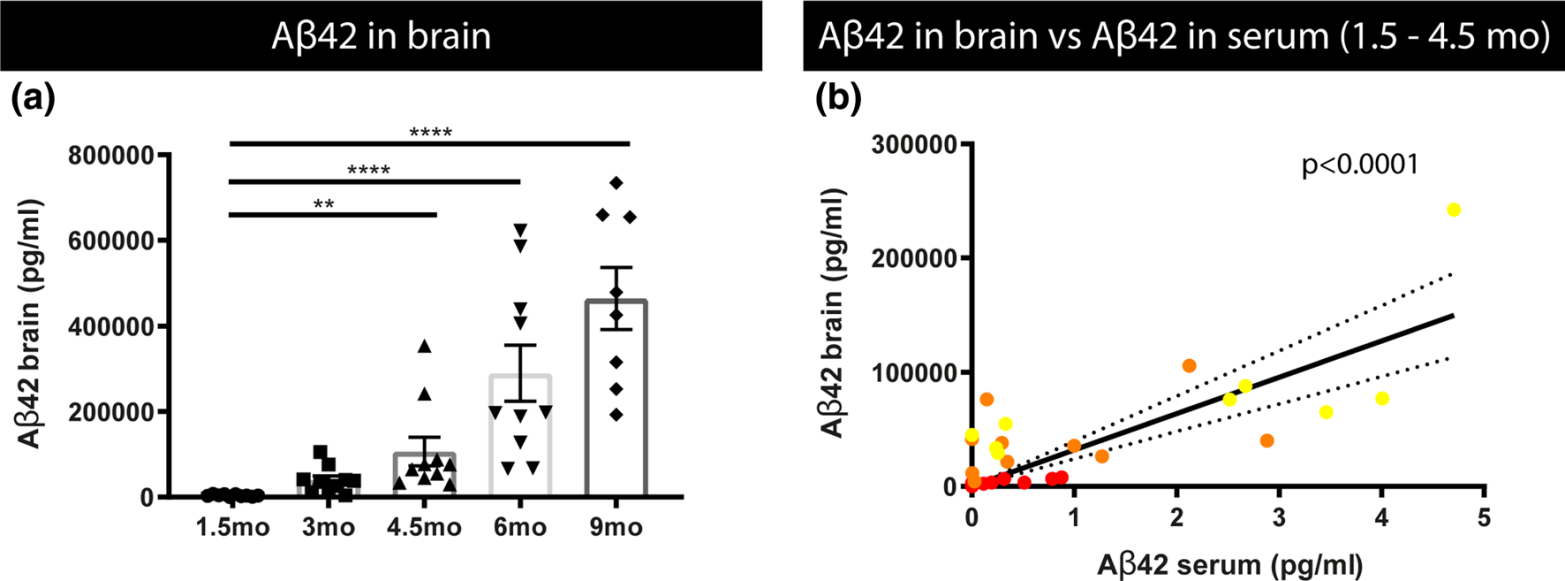
Relation between Aβ42 concentrations of 5xFAD mice in brain and serum. **a** Brain Aβ42 concentrations were measured in total brain extracts using electrochemiluminescence detecting Aβ42 in the different age groups of 5xFAD mice. Quantitative analysis revealed a significant increase in 4.5-month-, 6-month- and 9-month-old mice compared to 1.5-month-old mice. Kruskal–Wallis test with Dunn’s multiple comparison (non‐normally distributed). Data are presented as means ± SEM; *p < 0.05; **p < 0.01; ***p < 0.001; ****p < 0.0001 (1.5 months, 3 months, 4.5 months, 6 months and 9 months: *n* = 10, 10, 10, 10, 8; for all groups: *n* = 8–10). **b)** Spearman´s correlation analysis between brain (FrCx) and serum Aβ42 levels at 1.5 (red), 3 (orange) and 4.5 (yellow) months old in 5XFAD mice (*r*_s_ = 0.6304, *p* < 0.0005, *n* = 29). Linear regression analysis demonstrated a positive slope significantly different from 0 (*p* < 0.0001)

To further assess the relation between the Aβ concentrations in serum with brain-related processes in AD, we assessed their relation with Aβ concentrations in brain homogenates. Concentrations of soluble Aβ42 were measured in total brain homogenates of 5xFAD mice of all age groups using electrochemiluminescence-based assay detecting Aβ42. This demonstrated significantly increased Aβ42 concentrations in total brain homogenates of 4.5-month-old compared to 1.5-month-old 5xFAD mice (Fig. 7). This is in line with accumulating Aβ concentrations reaching threshold values for aggregation and plaque formation. We further assessed correlations between Aβ42 serum concentrations and Aβ42 concentrations in total brain homogenates. Linear regression analysis demonstrated a positive slope significantly different from 0 (*p*-value < 0.0001) (Fig. 7b). Furthermore, Spearman correlation analysis demonstrated a significant and strong positive correlation (*p*-value < 0.0005, *r*_s_ = 0.6304) between Aβ42 serum concentrations and Aβ42 concentrations in brain in the earliest pre-pathological phase. Also for Aβ40 linear regression and Spearman’s correlation revealed a significant correlation between BB-Aβ40 concentrations and increasing Aβ40 concentrations in the brain between 1.5 and 4.5 months, in this early pre-pathological phase (*r*_s_ = 0.489, *p* < 0.05) (Supplementary Fig. S4, online resource). Our data indicate that increasing BB-Aβ, BB-Aβ42 and BB-Aβ40, correlate with increasing Aβ-concentrations, Aβ42 and Aβ40, in the brain in the earliest pre-pathological phase. These data highlight the potential of BB-Aβ concentrations, as biomarker for tracking increasing concentrations of Aβ in the brain in the early pre-pathological phase. Our data thereby may open new avenues for using BB-Aβ as indicators of accumulating Aβ in the brain.

### Longitudinal analysis of serum Aβ42 reveals a biphasic pattern, with increasing Aβ42 concentrations in the earliest pre-pathological stage

**Fig. 8:**
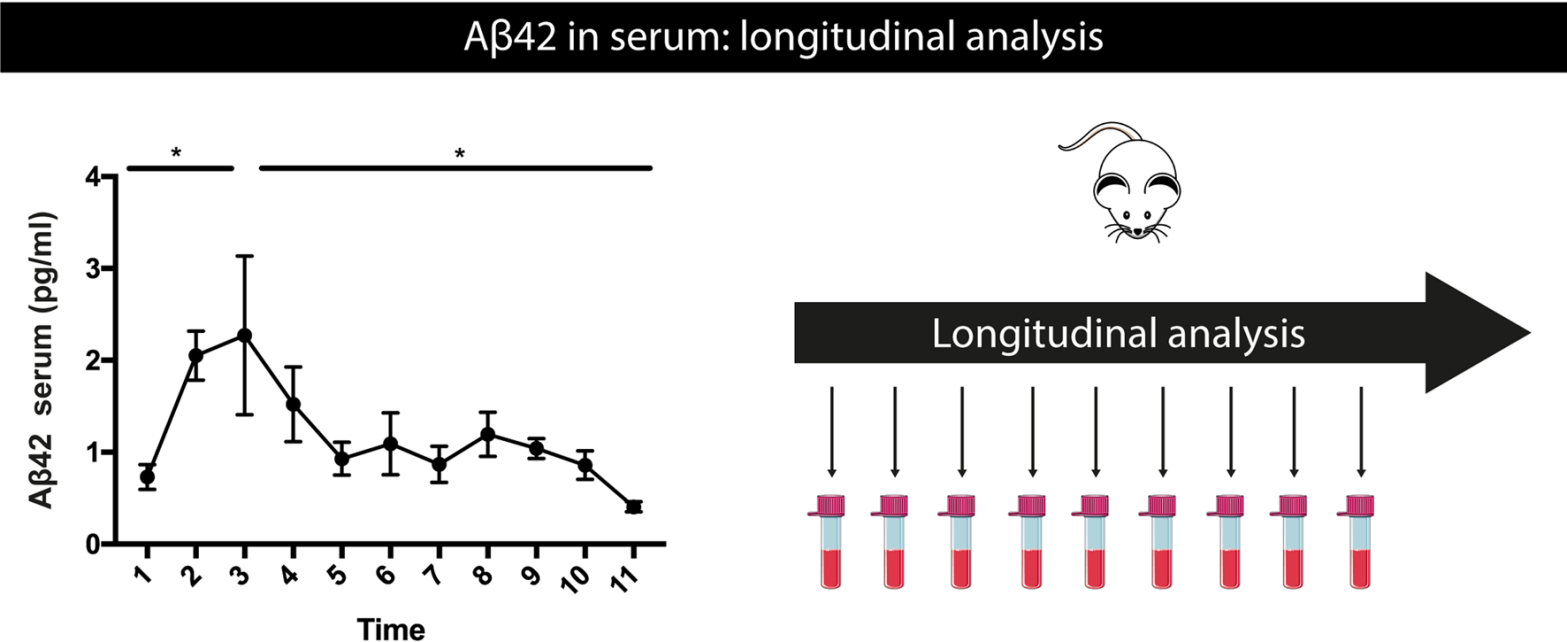
Longitudinal dynamics of serum Aβ42 in 5xFAD mice. Quantitative analysis of Aβ42 concentrations in serum using electrochemiluminescence assay, of longitudinally collected serum samples (11 time-points were collected, starting from 2.5 months onwards), reveals an initial significant increase followed by a significant decrease in Aβ42 serum concentrations. This reveals biphasic dynamics of Aβ42 concentrations in 5xFAD mice. Repeated measures one-way ANOVA with Dunnett’s multiple comparison (*n* = 5). Missing samples (*n* = 5/55) were filled in using the mean of values. Data are presented as mean ± SEM; *p < 0.05; **p < 0.01; ***p < 0.001; ****p < 0.0001

Preclinical models provide several assets for biomarker analysis, including the analysis of the pre-pathological phase, as performed here, but also facilitating longitudinal analysis of biomarkers in relation to brain-related processes. We hence complemented the above cross-sectional analysis with a longitudinal analysis, assessing individual changes per mouse in an age-dependent manner, by collecting triweekly blood samples. Longitudinal analysis indicated a significant increase in serum Aβ42 concentrations in the initial pre-pathological phase, before significant robust amyloid plaque formation (Fig. 8). Longitudinal Aβ42 serum measurements furthermore confirmed a biphasic modulation of Aβ42 concentrations in serum in an age-dependent way. This supports increasing concentrations in BB-Aβ42, followed by a subsequent decrease in BB-Aβ42. Analysis of Aβ40 indicated a significant increase in the early pre-pathological phase, while no significant decrease was found in the pathological phase (Supplementary Fig. S7, online resource). No significant changes were found in Aβ42/Aβ40 ratio in the pre-pathological phase, while significantly decreased in the pathological stage (Supplementary Fig. S7, online resource). All mice of the longitudinal analysis displayed robust amyloid pathology (data not shown) at the age of dissection. While some variations are observed between longitudinal and cross-sectional analysis, which could be ascribed to differences in blood sampling (i.e., cardiac puncture vs. facial vein), as well as multiple sequential sampling of the same mice in the longitudinal analysis, strikingly similar findings regarding dynamics of BB-Aβ42, BB-Aβ40 and BB-Aβ42/Aβ40 were obtained in both analyses. Based on the combined cross-sectional analysis and longitudinal analysis, we find that BB-Aβ (BB-Aβ40 and BB-Aβ42) concentration initially increases in serum in the pre-pathological phase, correlating with increasing or accumulating Aβ concentrations in the brain, which could enable reaching a critical threshold concentration for amyloid plaque development. This initial phase is followed by robust amyloid deposition, characterized by decreasing Aβ42 concentrations in serum.

Taken together our analysis in a preclinical model recapitulating amyloid pathology, using longitudinal and cross-sectional analyses, indicates biphasic Aβ42 dynamics, characterized by a rise before decreasing Aβ42 concentrations in serum. This increase in serum reflects increasing Aβ concentrations in brain, also observed for BB-Aβ40, in the pre-pathological phase. This is in line with the hypothesis of increasing Aβ concentrations in brain enabling to reach threshold concentrations for Aβ aggregation. This early phase presents an important preventive window to interfere with increasing Aβ concentrations in the brain, before robust amyloid pathology is developing. Concomitant BB-Aβ monitoring, combined with intervention by life-style factors or risk factor prevention, may open new avenues for preventive personalized strategies, to prevent development of amyloid pathology, in the earliest pre-pathological stage.

### Aβ concentrations in plasma increase in older compared to young non-AD human subjects

**Fig. 9:**
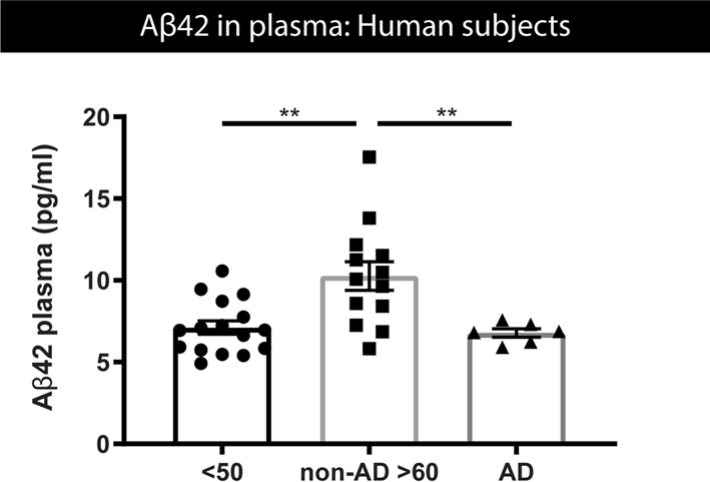
Aβ42 concentrations in plasma of human subjects. Measurements of Aβ42 concentrations in plasma of human subjects, measured using SIMOA. Quantitative analysis reveals a significant higher Aβ42 plasma concentration in > 60 years controls, compared to age-matched > 60 years AD patients, as well as compared to < 50 years controls. One‐way ANOVA with Dunnett’s multiple comparison test (normally distributed) (controls < 50 years, *n* = 16; controls > 60 years, *n* = 13; AD patients *n* = 6). Data are presented as mean ± SEM; *p < 0.05; **p < 0.01; ***p < 0.001; ****p < 0.0001

To further evaluate our findings in human subjects, we first assessed Aβ42 concentrations in plasma of human subjects. Blood samples were collected from individuals of a cohort with human subjects with different ages ranging from 20 to 50 years and individuals above 60 years, as well as AD patients [Fig. 9, Supplementary Fig. S8, online resource (table demographics)]. Plasma was collected and used for measurement of Aβ42 concentrations, using SIMOA for Aβ42. Importantly, this indicated a significant increase in BB-Aβ42 in individuals > 60 years compared to Aβ42 concentrations in individuals of < 50 years (Fig. 9). Furthermore, Aβ42 concentrations in plasma of AD patients was significantly lower compared to age-matched control subjects. These data further support our findings obtained in a preclinical model, indicating a biphasic pattern of blood-based Aβ42 concentrations in the earliest pre-pathological phase, in human subjects (Fig. 9). A similar biphasic pattern was found for Aβ42 in CSF (Supplementary Fig. S10, online resource) in this cohort. Furthermore, concomitant analysis of Aβ40 in this SIMOA assay, revealed a significant increase of plasma Aβ40 in older control subjects (> 60 years) compared to young (< 50 years), while no significant change was found between AD patients and age-matched control subjects (Supplementary Fig. S9, online resource). The ratio of plasma Aβ42/Aβ40 was not significantly different between young and older subjects, while AD patients displayed a significantly lower Aβ42/Aβ40 ratio compared to age-matched control subjects, in line with previous findings regarding plasma Aβ42/Aβ40 ratio in the pathological stage (Supplementary Fig. S9, online resource).

We further assessed correlation between plasma and CSF measurements in our cohort. Pearson’s correlation analysis and linear regression demonstrated a significant correlation between Aβ42 in plasma and Aβ42 in CSF (*r* = 0.396, *p* = 0.022) and Aβ42 in plasma and Aβ42/Aβ40 ratio in CSF (*r* = 0.525, *p* = 0.0024) (Supplementary Fig. S10, online resource). Linear regression analysis revealed a significant correlation between Aβ40 in plasma and CSF (*p* < 0.001), while Pearson’s correlation analysis was not significant. Linear regression analysis between t-tau in plasma and in CSF (*p* < 0.001), also revealed a significant correlation, but no significant correlation was demonstrated using Pearson’s correlation analysis. In this respect, it must be noted, that we here used the Quanterix t-tau assay, which was shown to be outperformed by other t-tau assays [48] for correlations between t-tau in plasma and CSF. Furthermore, only a small cohort is used here, with only limited number of AD cases, which is a limitation of the current study. It must also be noted that the control subjects in this cohort may include subjects that will develop AD or other neurological diseases or conversely will not develop AD nor other neurological diseases at late age. And ApoE4 status is not available for the complete cohort, hence not reported. Despite these limitations, revealing the importance of follow-up studies also including larger cohorts and longitudinal studies, we here corroborate previous findings in the pathological stage regarding Aβ and findings in our preclinical model. Most importantly, in this human cohort, we show changes in plasma in the early pre-pathological stage for Aβ42 and Aβ40, which strongly corroborate and extend our findings from a preclinical model regarding Aβ to a human cohort.

Our data in this human cohort confirm increased Aβ42 in plasma in aged compared to young controls, as well as compared to age-matched AD patients. This reveals a biphasic dynamic for BB-Aβ42 in a human cohort, further corroborated by biphasic dynamic in CSF-Aβ42. Furthermore, we demonstrate increasing plasma Aβ (Aβ42 and Aβ40) with aging in this human cohort, in line with increasing BB-Aβ (BB-Aβ42 and BB-Aβ40) in the early pre-pathological stage in our preclinical amyloid model. Our data provide a basis for further research, to assess the importance of measuring blood-based AD biomarkers particularly Aβ40 and Aβ42 in the earliest pre-pathological stages of AD, as markers of pathological processes in the brain.

## Discussion

In this work, we used the robustness, predictability and experimental potential of preclinical models to study the relation between Aβ- and tau-related blood-based biomarkers, Aβ and tau pathology and Aβ- and tau-related pathological processes, including increasing concentrations, phosphorylation and aggregation in the brain. We here corroborate the validity of blood-based assessment of Aβ and tau as biomarkers, not only to reflect Aβ and tau pathology, but also concentrations of pathological forms of Aβ and tau in the brain. We here demonstrate increasing BB-Aβ42 in the earliest pre-pathological phase, followed by decreasing BB-Aβ42 in the pathological phase, indicating a biphasic pattern. Most importantly, increasing concentrations of BB-Aβ, i.e., BB-Aβ42 and BB-Aβ0 in the early pre-pathological phase, correlate with increasing Aβ, i.e., Aβ42 and Aβ0 concentrations in the brain before robust amyloid pathology development, opening interesting perspectives for their use as biomarkers for preventive strategies in this pre-pathological phase. Finally, we validate these findings in plasma samples of a cohort of human subjects. Our data not only confirm previous findings concerning the pathological phase, corroborating the use of blood-based biomarkers in the pathological stages of AD, but most importantly, we here provide evidence for their value in the earliest pre-pathological stage, based on data in preclinical models and human subjects. We here demonstrate that increasing Aβ concentrations reflect increasing Aβ concentrations in the brain, in a critical window for prevention of formation of amyloid pathology. Taken together our findings and further validation of Aβ-biomarkers in the earliest pre-pathological phase may open new avenues for personalized preventive therapies.

Based on compelling evidence of EOAD cases (< 1%), biofluid biomarkers stand as a promising strategy to predict/monitor AD progression [5, 9, 41]. Classically, the AD biomarker profile consists of a reduction of Aβ42 peptide, a low Aβ42/40 ratio, as well as an increase in the levels of total tau and phosphorylated tau in cerebrospinal fluid (CSF) [33]. This profile, complemented with neuroimaging, is robustly recapitulated in sporadic AD cases (> 99%) thereby serving as diagnostic markers [61]. Compelling evidence supports their use as proxies for the ongoing pathological state in the brain. CSF Aβ42 and Aβ42 postmortem ventricular levels correlate with plaque load at autopsy [33, 53]. In patients robust evidence shows the relation between amyloid load as assessed by Pittsburgh Compound B (PIB)-PET binding and CSF Aβ42, with higher 11CPIB binding correlating with lower CSF Aβ42 levels [10, 58]. Additionally, CSF t-tau is shown to increase in acute disorders such as stroke, and this increase is proportional to the damage severity [35, 54, 63]. Furthermore, CSF levels of p-tau-Thr181 and p-tau-Thr231 correlate with neocortical tangle pathology [55]. Based on an extensive meta-analysis by Olsson and colleagues, t-tau, p-tau, NFL and Aβ42, were indicated for use in clinical practice and clinical research [33]. Taken together, these data support the role of CSF biomarkers as a reliable reflector of ongoing brain pathology. However, crucial factors, such as invasiveness and economical costs, pose scalability issues for routinely clinical practice for prevention strategies and to cope with future disease impact projections [1].

In this respect, AD-related blood-based biomarkers open interesting possibilities by providing affordable, non-invasive, and easy scalable methods. Emerging ultrasensitive immunoassay technologies like SIMOA platforms [42] have led to major breakthroughs in AD blood-based biomarkers field [31, 36–39, 46, 59, 60]. APP 669-711/Aβ1-42 and Aβ1-40/Aβ1-42 ratios have shown prediction potential for determination of amyloid-β PET status [31]. Moreover, low plasma Aβ42/Aβ40 correlates with low CSF Aβ42 and presence of amyloid pathology in the brain [59], suggesting the implication of plasma Aβ42/Aβ40 as an early diagnostic marker with clinical predictive value. Complementary, plasma t-tau shows to be a promising prognostic marker also for non-specific cognitive decline cases [27, 30, 39]. Also, plasma t-tau and p-tau was linked to rapid AD progression in later stages correlating with brain atrophy, hypo-metabolism and cognitive decline [27, 29, 36, 51]*.* Olsson and colleagues, identified a strong association between plasma t-tau and AD [33]. Additionally, plasma p-tau alone or in combination with other markers like brief cognitive tests and APOE genotype, has shown clear advantage for diagnosis and prediction of progression [38, 47].

A major bottleneck in AD diagnosis dwells on the inscalability of the CSF collection. The aforementioned high impact and important studies represent the pavement toward future blood-based diagnostic modalities, while in-depth insights between BB biomarkers and AD-related processes in brain may support and increase their further use. In this work we took advantage of well-characterized preclinical models, with well-known spatio-temporal development of pathology to gain insights in biomarker dynamics in the earliest pre-pathological phase. We here show positive correlation for BB-tau increasing with increasing development of tau pathology in a preclinical model. We furthermore show correlations between BB-tau and increasing p-tau-Thr231 and tau aggregates in the brain in tau transgenic mice. In this study, serum-based biomarkers were used, and hence clotting must be considered, and consequently follow-up studies should consider including plasma-based analysis, as well as the use of different preclinical models. However, a recent study validated the use of both serum and plasma for BB-tau analysis, to reflect tau pathology in humans [17]. Furthermore, our findings regarding Aβ are validated using analysis in plasma in our human cohort.

Importantly, in this work, we show biphasic BB-Aβ42-dynamics, with BB-Aβ42 initially increasing, followed by a decrease in the pathological stage. Notably, increased BB-Aβ in the earliest pre-pathological stage correlated with accumulating Aβ in the brain, supporting its utility as biomarker, with interesting implications for prevention strategies, based on BB biomarkers, enabling scalability and flexibility that are essential for prevention strategies. Previous studies measuring CSF in well-characterized preclinical models with well-characterized spatio-temporal pathology development, have indicated a biphasic modulation of CSF-Aβ [26]. Indeed, CSF Aβ was shown to be increased with amyloid pathology, in the pre-pathological stage [26], and a biphasic pattern was shown for CSF Aβ42 in a preclinical model. The combined data are in line with the hypothesis that Aβ concentrations in brain may increase to reach a threshold concentration enabling Aβ and tau aggregation. This hypothesis is also in line with our findings in our preclinical amyloid model, indicating increasing Aβ concentrations in the brain (Aβ40 and Aβ42). We furthermore also showed a biphasic Aβ42 dynamics in CSF in human subjects, characterized by an early increase in CSF Aβ42 in the earliest pre-pathological phase, from young (< 50) to older (< 60) in line with the findings of Maia et al. for Aβ42 in preclinical models [26]. These data are furthermore in line with findings in a human cohort showing that in ApoE4 negative subjects (males and females), CSF Aβ42 was predicted to increase between 25 and 50 years of age based on linear regression of CSF Aβ42 on the three-way age x gender x APOE genotype interaction [23]. Most importantly, we here now not only extend these data to increasing BB-Aβ concentrations in the earliest pre-pathological stage, but also their correlation with increasing concentration of Aβ in the brain in a preclinical amyloid model, extending insights in the pre-pathological phase to scalable and flexible BB biomarkers. We further validated our findings regarding BB-Aβ in plasma samples of a small human cohort, demonstrating decreased plasma Aβ42 in AD patients compared to non-AD, and an increase in plasma Aβ42 in older compared to younger control subjects, indicating a biphasic pattern. Our data are in line with previous data demonstrating decreased Aβ42 plasma concentrations in AD patients compared to control in the pathological stage, but reveal innovative insights in flexible BB biomarkers in the pre-pathological stages. A similar increase was demonstrated for BB-Aβ40 in this pre-pathological stage in our human cohort. We believe our data provide novel insights in dynamics of biomarkers in relation to pathogenic AD processes in the brain, particularly in the pre-pathological stage, opening new perspectives for prevention strategies.

## Conclusion

Here we analyzed dynamics between Aβ and tau-related blood-based biomarkers and Aβ and tau-related pathological processes in the brain, using preclinical models and human subjects. We show that serum tau levels correlated with tau pathology, and with accumulation of p-tau and tau aggregation measured biochemically in the brain. We furthermore confirm decreased Aβ42 concentrations with progressive robust amyloid pathology, in line with previous findings. Importantly, we here demonstrated an initial increase of BB-Aβ42 followed by a subsequent decrease in BB-Aβ42, in a preclinical model and human cohort, indicating biphasic dynamics of BB-Aβ42. Most importantly, we here show an increase in BB-Aβ, i.e., Aβ42 and Aβ40 in the earliest pre-pathological phase, both in a preclinical model and human cohort. Notably, increasing BB-Aβ —Aβ42 and Aβ40— correlated with increasing Aβ concentrations —Aβ42 and Aβ40— in total brain homogenates, and with increasing initial pathology in this phase, in a preclinical model. Our data herewith open new avenues for preventive strategies in this earliest phase. We believe our data provide a basis for the further study and use of blood-based biomarkers in this earliest phase as biomarkers and proxies for increasing Aβ concentrations in the brain, opening novel potential avenues for assessing effect of lifestyle and risk interventions. While further detailed analysis is required, the presented data provide interesting novel perspectives for personalized preventive strategies for AD, as prevention of AD in the earliest pre-pathological stage, presents as an important target for this irreversible, devastating, progressive de-humanizing process.

## Supplementary Information

Below is the link to the electronic supplementary material.

Supplementary file1 (PDF 97967 kb)

## References

[1] (2020) 2020 Alzheimer's disease facts and figures. Alzheimers Dement: Doi 10.1002/alz.12068 10.1002/alz.12068 32157811

[2] Albert MS, DeKosky ST, Dickson D, Dubois B, Feldman HH, Fox NC (2011). The diagnosis of mild cognitive impairment due to Alzheimer's disease: recommendations from the National Institute on Aging-Alzheimer's Association workgroups on diagnostic guidelines for Alzheimer's disease. Alzheimers Dement.

[3] Ashton NJ, Pascoal TA, Karikari TK, Benedet AL, Lantero-Rodriguez J, Brinkmalm G (2021). Plasma p-tau231: a new biomarker for incipient Alzheimer's disease pathology. Acta Neuropathol.

[4] Barthelemy NR, Li Y, Joseph-Mathurin N, Gordon BA, Hassenstab J, Benzinger TLS (2020). A soluble phosphorylated tau signature links tau, amyloid and the evolution of stages of dominantly inherited Alzheimer's disease. Nat Med.

[5] Bateman RJ, Xiong C, Benzinger TL, Fagan AM, Goate A, Fox NC (2012). Clinical and biomarker changes in dominantly inherited Alzheimer's disease. N Engl J Med.

[6] Braak H, Braak E (1991). Neuropathological stageing of Alzheimer-related changes. Acta Neuropathol.

[7] Chhatwal JP, Schultz AP, Dang Y, Ostaszewski B, Liu L, Yang HS (2020). Plasma N-terminal tau fragment levels predict future cognitive decline and neurodegeneration in healthy elderly individuals. Nat Commun.

[8] Dubois B, Villain N, Frisoni GB, Rabinovici GD, Sabbagh M, Cappa S (2021). Clinical diagnosis of Alzheimer's disease: recommendations of the International Working Group. Lancet Neurol.

[9] Gordon BA, Blazey TM, Su Y, Hari-Raj A, Dincer A, Flores S (2018). Spatial patterns of neuroimaging biomarker change in individuals from families with autosomal dominant Alzheimer's disease: a longitudinal study. Lancet Neurol.

[10] Grimmer T, Riemenschneider M, Forstl H, Henriksen G, Klunk WE, Mathis CA (2009). Beta amyloid in Alzheimer's disease: increased deposition in brain is reflected in reduced concentration in cerebrospinal fluid. Biol Psychiatry.

[11] Hampel H, O'Bryant SE, Molinuevo JL, Zetterberg H, Masters CL, Lista S (2018). Blood-based biomarkers for Alzheimer disease: mapping the road to the clinic. Nat Rev Neurol.

[12] Hanseeuw BJ, Betensky RA, Jacobs HIL, Schultz AP, Sepulcre J, Becker JA (2019). Association of amyloid and tau with cognition in preclinical Alzheimer disease: a longitudinal study. JAMA Neurol.

[13] Hansson O (2021). Biomarkers for neurodegenerative diseases. Nat Med.

[14] Jack CR, Bennett DA, Blennow K, Carrillo MC, Dunn B, Haeberlein SB (2018). NIA-AA Research Framework: Toward a biological definition of Alzheimer's disease. Alzheimers Dement.

[15] Janelidze S, Mattsson N, Palmqvist S, Smith R, Beach TG, Serrano GE (2020). Plasma P-tau181 in Alzheimer's disease: relationship to other biomarkers, differential diagnosis, neuropathology and longitudinal progression to Alzheimer's dementia. Nat Med.

[16] Janelidze S, Palmqvist S, Leuzy A, Stomrud E, Verberk IMW, Zetterberg H (2021). Detecting amyloid positivity in early Alzheimer's disease using combinations of plasma Abeta42/Abeta40 and p-tau. Alzheimers Dement.

[17] Kac PR, Gonzalez-Ortiz F, Simren J, Dewit N, Vanmechelen E, Zetterberg H (2022). Diagnostic value of serum versus plasma phospho-tau for Alzheimer's disease. Alzheimers Res Ther.

[18] Kaeser SA, Hasler LM, Lambert M, Bergmann C, Bottelbergs A, Theunis C (2022). CSF p-tau increase in response to Abeta-type and Danish-type cerebral amyloidosis and in the absence of neurofibrillary tangles. Acta Neuropathol.

[19] Kawarabayashi T, Younkin LH, Saido TC, Shoji M, Ashe KH (2001). Age-dependent changes in brain, CSF, and plasma amyloid (beta) protein in the Tg2576 transgenic mouse model of Alzheimer's disease. J Neurosci.

[20] Kim K, Kim MJ, Kim DW, Kim SY, Park S, Park CB (2020). Clinically accurate diagnosis of Alzheimer's disease via multiplexed sensing of core biomarkers in human plasma. Nat Commun.

[21] Lantero Rodriguez J, Karikari TK, Suarez-Calvet M, Troakes C, King A, Emersic A (2020). Plasma p-tau181 accurately predicts Alzheimer's disease pathology at least 8 years prior to post-mortem and improves the clinical characterisation of cognitive decline. Acta Neuropathol.

[22] Leuzy A, Cullen NC, Mattsson-Carlgren N, Hansson O (2021). Current advances in plasma and cerebrospinal fluid biomarkers in Alzheimer's disease. Curr Opin Neurol.

[23] Li G, Shofer JB, Petrie EC, Yu CE, Wilkinson CW, Figlewicz DP (2017). Cerebrospinal fluid biomarkers for Alzheimer's and vascular disease vary by age, gender, and APOE genotype in cognitively normal adults. Alzheimers Res Ther.

[24] Lodder C, Scheyltjens I, Stancu IC, Botella Lucena P, Gutierrez de Rave M, Vanherle S (2021). CSF1R inhibition rescues tau pathology and neurodegeneration in an A/T/N model with combined AD pathologies, while preserving plaque associated microglia. Acta Neuropathol Commun.

[25] Maia LF, Kaeser SA, Reichwald J, Hruscha M, Martus P, Staufenbiel M (2013). Changes in amyloid-beta and Tau in the cerebrospinal fluid of transgenic mice overexpressing amyloid precursor protein. Sci Transl Med.

[26] Maia LF, Kaeser SA, Reichwald J, Lambert M, Obermuller U, Schelle J (2015). Increased CSF Abeta during the very early phase of cerebral Abeta deposition in mouse models. EMBO Mol Med.

[27] Mattsson N, Zetterberg H, Janelidze S, Insel PS, Andreasson U, Stomrud E (2016). Plasma tau in Alzheimer disease. Neurology.

[28] McKhann GM, Knopman DS, Chertkow H, Hyman BT, Jack CR, Kawas CH (2011). The diagnosis of dementia due to Alzheimer's disease: recommendations from the National Institute on Aging-Alzheimer's Association workgroups on diagnostic guidelines for Alzheimer's disease. Alzheimers Dement.

[29] Meakin PJ, Coull BM, Tuharska Z, McCaffery C, Akoumianakis I, Antoniades C (2020). Elevated circulating amyloid concentrations in obesity and diabetes promote vascular dysfunction. J Clin Invest.

[30] Molinuevo JL, Ayton S, Batrla R, Bednar MM, Bittner T, Cummings J (2018). Current state of Alzheimer's fluid biomarkers. Acta Neuropathol.

[31] Nakamura A, Kaneko N, Villemagne VL, Kato T, Doecke J, Dore V (2018). High performance plasma amyloid-beta biomarkers for Alzheimer's disease. Nature.

[32] Oakley H, Cole SL, Logan S, Maus E, Shao P, Craft J (2006). Intraneuronal beta-amyloid aggregates, neurodegeneration, and neuron loss in transgenic mice with five familial Alzheimer's disease mutations: potential factors in amyloid plaque formation. J Neurosci.

[33] Olsson B, Lautner R, Andreasson U, Ohrfelt A, Portelius E, Bjerke M (2016). CSF and blood biomarkers for the diagnosis of Alzheimer's disease: a systematic review and meta-analysis. Lancet Neurol.

[34] Ossenkoppele R, Reimand J, Smith R, Leuzy A, Strandberg O, Palmqvist S (2021). Tau PET correlates with different Alzheimer's disease-related features compared to CSF and plasma p-tau biomarkers. EMBO Mol Med.

[35] Ost M, Nylen K, Csajbok L, Ohrfelt AO, Tullberg M, Wikkelso C (2006). Initial CSF total tau correlates with 1-year outcome in patients with traumatic brain injury. Neurology.

[36] Palmqvist S, Insel PS, Stomrud E, Janelidze S, Zetterberg H, Brix B (2019). Cerebrospinal fluid and plasma biomarker trajectories with increasing amyloid deposition in Alzheimer's disease. EMBO Mol Med.

[37] Palmqvist S, Janelidze S, Stomrud E, Zetterberg H, Karl J, Zink K (2019). Performance of fully automated plasma assays as screening tests for Alzheimer disease-related beta-amyloid status. JAMA Neurol.

[38] Palmqvist S, Tideman P, Cullen N, Zetterberg H, Blennow K, Alzheimer's Disease Neuroimaging I (2021). Prediction of future Alzheimer's disease dementia using plasma phospho-tau combined with other accessible measures. Nat Med.

[39] Pase MP, Beiser AS, Himali JJ, Satizabal CL, Aparicio HJ, DeCarli C (2019). Assessment of plasma total tau level as a predictive biomarker for dementia and related endophenotypes. JAMA Neurol.

[40] Pereira JB, Janelidze S, Stomrud E, Palmqvist S, van Westen D, Dage JL (2021). Plasma markers predict changes in amyloid, tau, atrophy and cognition in non-demented subjects. Brain.

[41] Reiman EM, Quiroz YT, Fleisher AS, Chen K, Velez-Pardo C, Jimenez-Del-Rio M (2012). Brain imaging and fluid biomarker analysis in young adults at genetic risk for autosomal dominant Alzheimer's disease in the presenilin 1 E280A kindred: a case-control study. Lancet Neurol.

[42] Rissin DM, Kan CW, Campbell TG, Howes SC, Fournier DR, Song L (2010). Single-molecule enzyme-linked immunosorbent assay detects serum proteins at subfemtomolar concentrations. Nat Biotechnol.

[43] Schelle J, Hasler LM, Gopfert JC, Joos TO, Vanderstichele H, Stoops E (2017). Prevention of tau increase in cerebrospinal fluid of APP transgenic mice suggests downstream effect of BACE1 inhibition. Alzheimers Dement.

[44] Scheltens P, Blennow K, Breteler MM, de Strooper B, Frisoni GB, Salloway S (2016). Alzheimer's disease. Lancet.

[45] Serrano-Pozo A, Frosch MP, Masliah E, Hyman BT (2011). Neuropathological alterations in Alzheimer disease. Cold Spring Harb Perspect Med.

[46] Shi L, Westwood S, Baird AL, Winchester L, Dobricic V, Kilpert F (2019). Discovery and validation of plasma proteomic biomarkers relating to brain amyloid burden by SOMAscan assay. Alzheimers Dement.

[47] Smirnov DS, Ashton NJ, Blennow K, Zetterberg H, Simren J, Lantero-Rodriguez J (2022). Plasma biomarkers for Alzheimer's disease in relation to neuropathology and cognitive change. Acta Neuropathol.

[48] Snellman A, Lantero-Rodriguez J, Emersic A, Vrillon A, Karikari TK, Ashton NJ (2022). N-terminal and mid-region tau fragments as fluid biomarkers in neurological diseases. Brain.

[49] Stancu IC, Cremers N, Vanrusselt H, Couturier J, Vanoosthuyse A, Kessels S (2019). Aggregated Tau activates NLRP3-ASC inflammasome exacerbating exogenously seeded and non-exogenously seeded Tau pathology in vivo. Acta Neuropathol.

[50] Stancu IC, Lodder C, Botella Lucena P, Vanherle S, Gutierrez de Rave M, Terwel D (2022). The NLRP3 inflammasome modulates tau pathology and neurodegeneration in a tauopathy model. Glia.

[51] Stancu IC, Ris L, Vasconcelos B, Marinangeli C, Goeminne L, Laporte V (2014). Tauopathy contributes to synaptic and cognitive deficits in a murine model for Alzheimer's disease. FASEB J.

[52] Stancu IC, Vasconcelos B, Ris L, Wang P, Villers A, Peeraer E (2015). Templated misfolding of Tau by prion-like seeding along neuronal connections impairs neuronal network function and associated behavioral outcomes in Tau transgenic mice. Acta Neuropathol.

[53] Strozyk D, Blennow K, White LR, Launer LJ (2003). CSF Abeta 42 levels correlate with amyloid-neuropathology in a population-based autopsy study. Neurology.

[54] Taghdiri F, Multani N, Tarazi A, Naeimi SA, Khodadadi M, Esopenko C (2019). Elevated cerebrospinal fluid total tau in former professional athletes with multiple concussions. Neurology.

[55] Tapiola T, Alafuzoff I, Herukka SK, Parkkinen L, Hartikainen P, Soininen H (2009). Cerebrospinal fluid {beta}-amyloid 42 and tau proteins as biomarkers of Alzheimer-type pathologic changes in the brain. Arch Neurol.

[56] Thal DR, Rub U, Orantes M, Braak H (2002). Phases of A beta-deposition in the human brain and its relevance for the development of AD. Neurology.

[57] Thomas KR, Bangen KJ, Edmonds EC, Weigand AJ, Walker KS, Bondi MW (2021). Objective subtle cognitive decline and plasma phosphorylated tau181: early markers of Alzheimer's disease-related declines. Alzheimers Dement (Amst).

[58] Tolboom N, van der Flier WM, Yaqub M, Boellaard R, Verwey NA, Blankenstein MA (2009). Relationship of cerebrospinal fluid markers to 11C-PiB and 18F-FDDNP binding. J Nucl Med.

[59] Verberk IMW, Slot RE, Verfaillie SCJ, Heijst H, Prins ND, van Berckel BNM (2018). Plasma amyloid as prescreener for the earliest Alzheimer pathological changes. Ann Neurol.

[60] Verberk IMW, Thijssen E, Koelewijn J, Mauroo K, Vanbrabant J, de Wilde A (2020). Combination of plasma amyloid beta(1–42/1-40) and glial fibrillary acidic protein strongly associates with cerebral amyloid pathology. Alzheimers Res Ther.

[61] Villemagne VL, Burnham S, Bourgeat P, Brown B, Ellis KA, Salvado O (2013). Amyloid beta deposition, neurodegeneration, and cognitive decline in sporadic Alzheimer's disease: a prospective cohort study. Lancet Neurol.

[62] Yoshiyama Y, Higuchi M, Zhang B, Huang SM, Iwata N, Saido TC (2007). Synapse loss and microglial activation precede tangles in a P301S tauopathy mouse model. Neuron.

[63] Zetterberg H, Hietala MA, Jonsson M, Andreasen N, Styrud E, Karlsson I (2006). Neurochemical aftermath of amateur boxing. Arch Neurol.

