# Supplementary material for: Blood-based Aβ42 increases in the earliest pre-pathological stage before decreasing with progressive amyloid pathology in preclinical models and human subjects: opening new avenues for prevention
Source: Acta Neuropathol. 2022 Jul 7;144(3):489–508. doi: 10.1007/s00401-022-02458-9 (PMC9381631; doi:10.1007/s00401-022-02458-9)
Supplement: Supplementary file 1 — Supplementary file1 (PDF 97967 kb) [file 401_2022_2458_MOESM1_ESM.pdf]

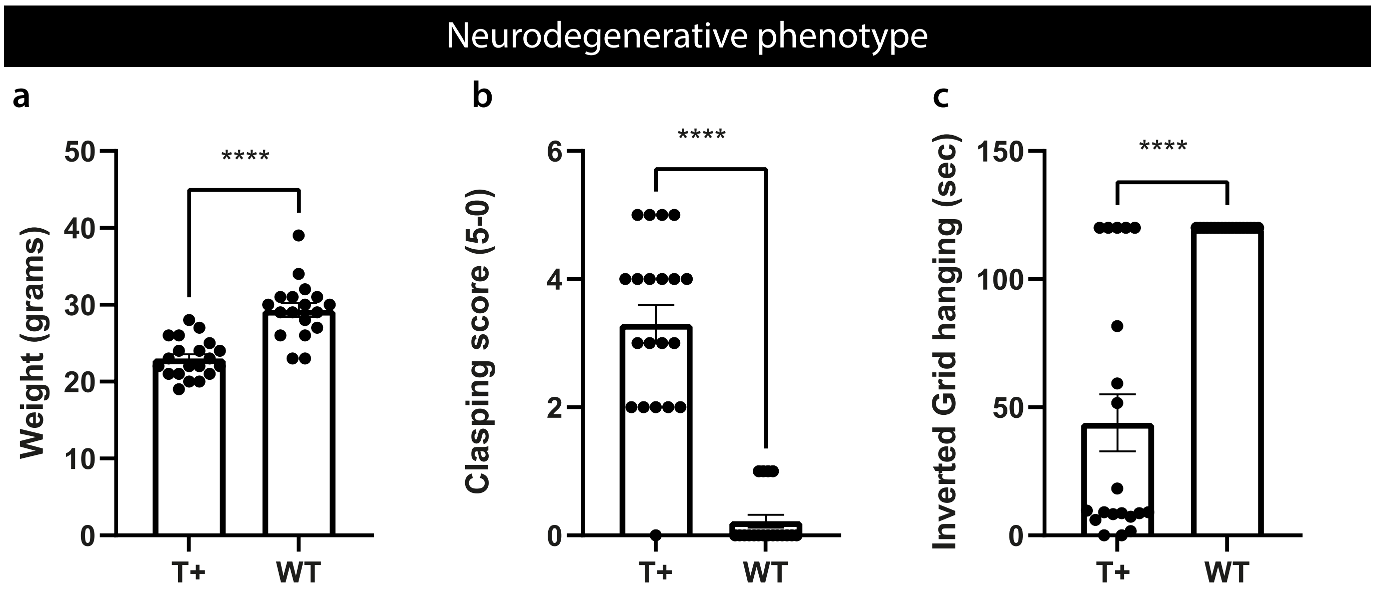

Figure S1. Progressive neurodegenerative phenotype in TauP301S (PS19) transgenic mice. Tau P301S (PS19) mice start displaying **(a)** weight loss, **(b)** clasping phenotype and **(c)** motor deficits from 11 to 12 months onwards.

Figure S1

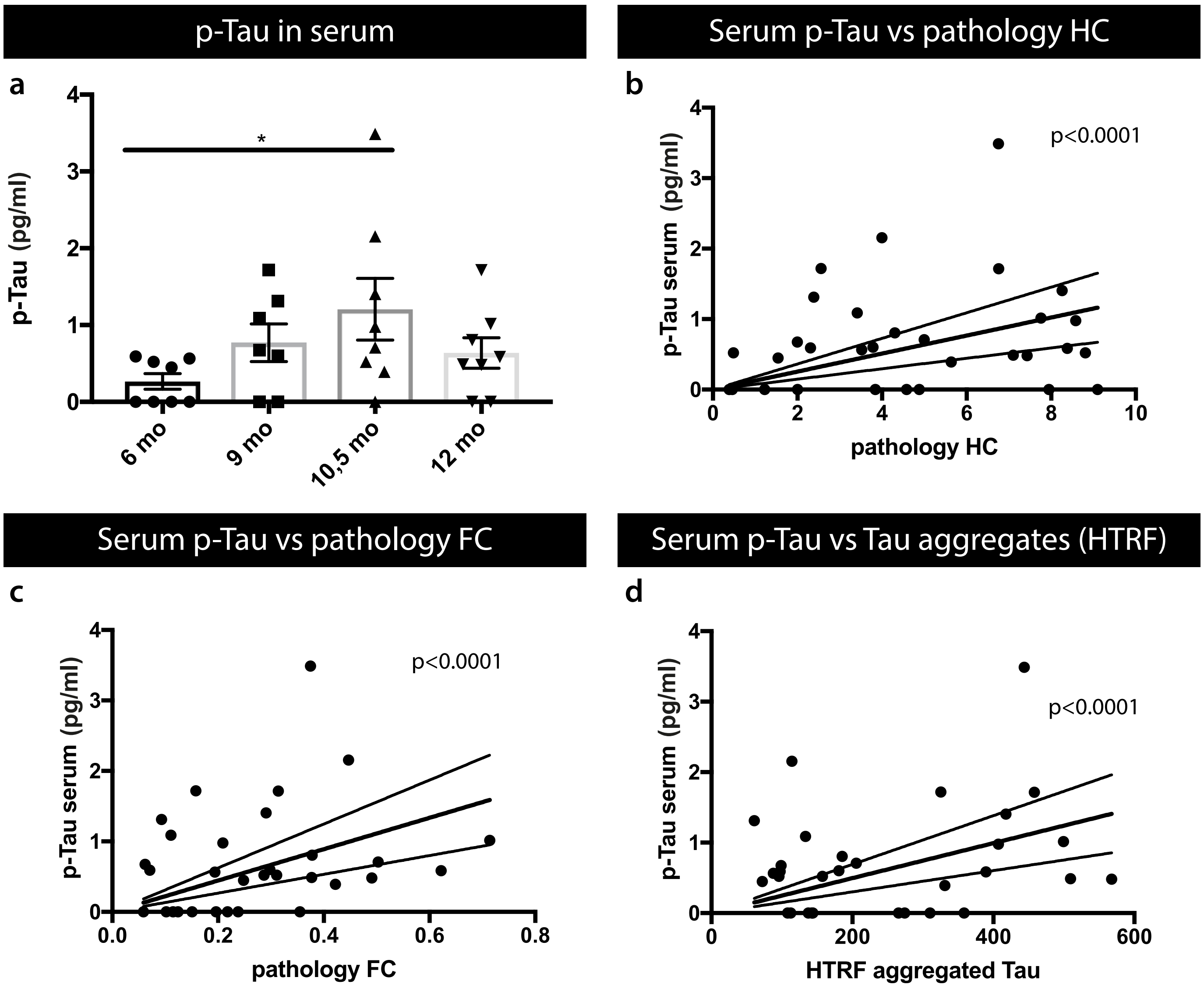

Figure S2 p-tau serum concentrations in relation to tau pathology in P301S mice
a) P-tau-Thr231 concentrations in serum of 6 mo, 9 mo, 10.5 mo and 12 mo old Tau mice were measured using electrochemiluminescence assay, revealing an age-dependent increase, being significantly increased at 10.5 months compared to 6 months of age. One-way ANOVA with Dunnett's multiple comparison test (normally distributed) **b), c), d)** Linear regression analysis demonstrated that the slope was significantly different from zero for p-tau-Thr231 serum levels in relation to tau pathology in HC ($p < 0.0001$) **(b)**, BS ($p < 0.01$) and FrCx ($p < 0.0001$) **(c)**, and in relation to p-tau-Thr231 concentration ($p < 0.0001$)) and tau-aggregates measured with HTRF in brain ($p < 0.0001$) **(d)**. **(a)** (6 mo: n=8; 9 mo: n=7; 10.5, 12 mo: n=8), 2 outliers ROUT 0,2%. Data are presented as means ± SEM; *$p < 0.05$; **$p < 0.01$; ***$p < 0.001$; ****$p < 0.0001$

Figure S2

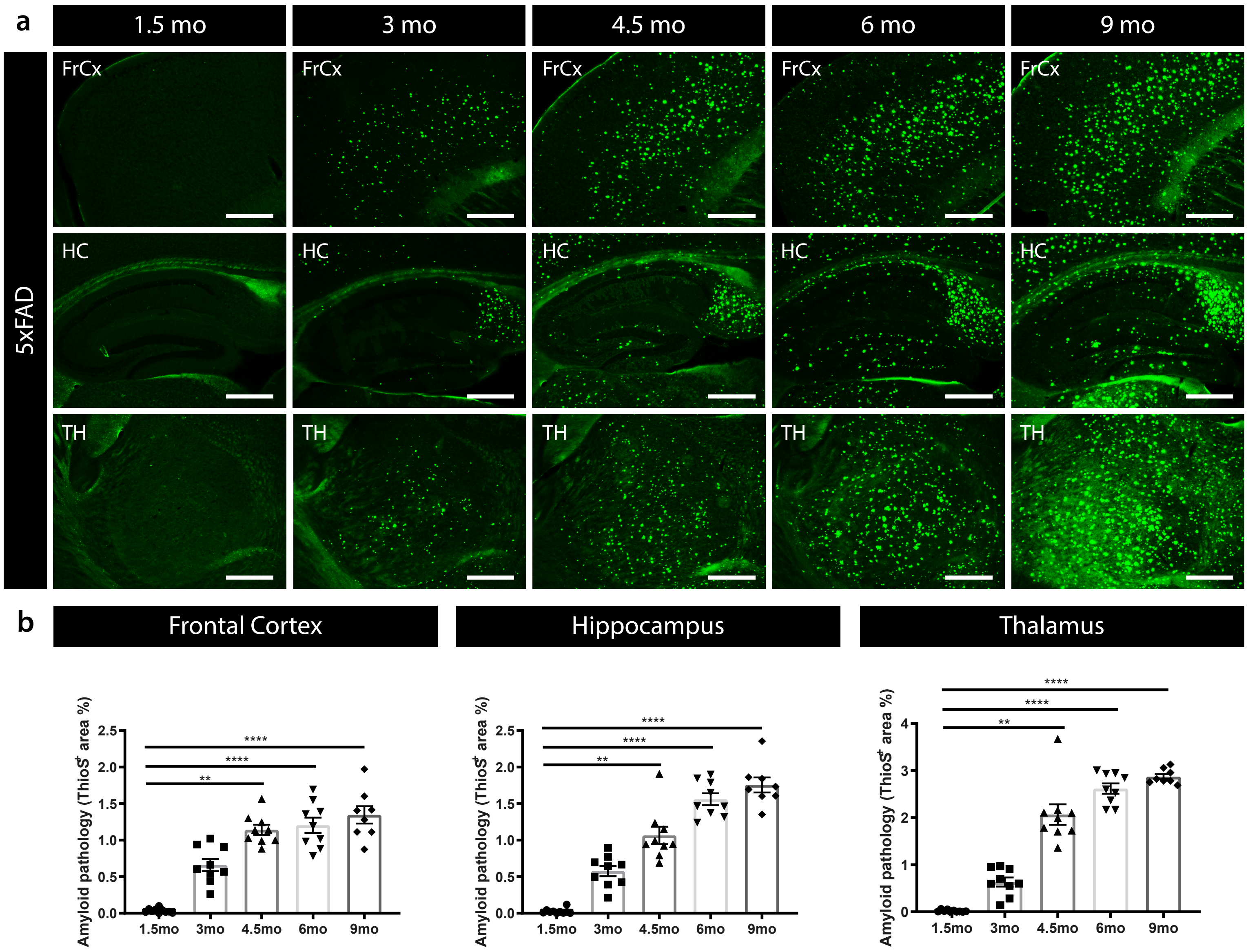

Figure S3. Development of amyloid pathology in 5xFAD mice using ThioS staining **a)** Representative images of ThioS staining in hippocampus, frontal cortex, and thalamus of APP/PS1 at 1.5 mo, 3 mo, 4.5 mo, 6 mo and 9 mo. **b)** Quantitative analysis of ThioS positive signal reveals a significant increase in signal starting at 4.5 months of age in FrCx, HC and TH. One-way ANOVA with Tukey's multiple comparison test (normally distributed); Kruskal–Wallis test with Dunn's multiple comparison (non-normally distributed). Data are presented as mean ± SEM; *$p < 0.05$; **$p < 0.01$; ***$p < 0.001$; ****$p < 0.0001$. (FrCx = Frontal Cortex; HC = Hippocampus; TH = Thalamus) (1.5 mo, 3 mo, 4.5 mo, 6 mo and 9 mo: n= 10, 9, 9, 9, 8; for all groups: n=8-10)

Figure S3

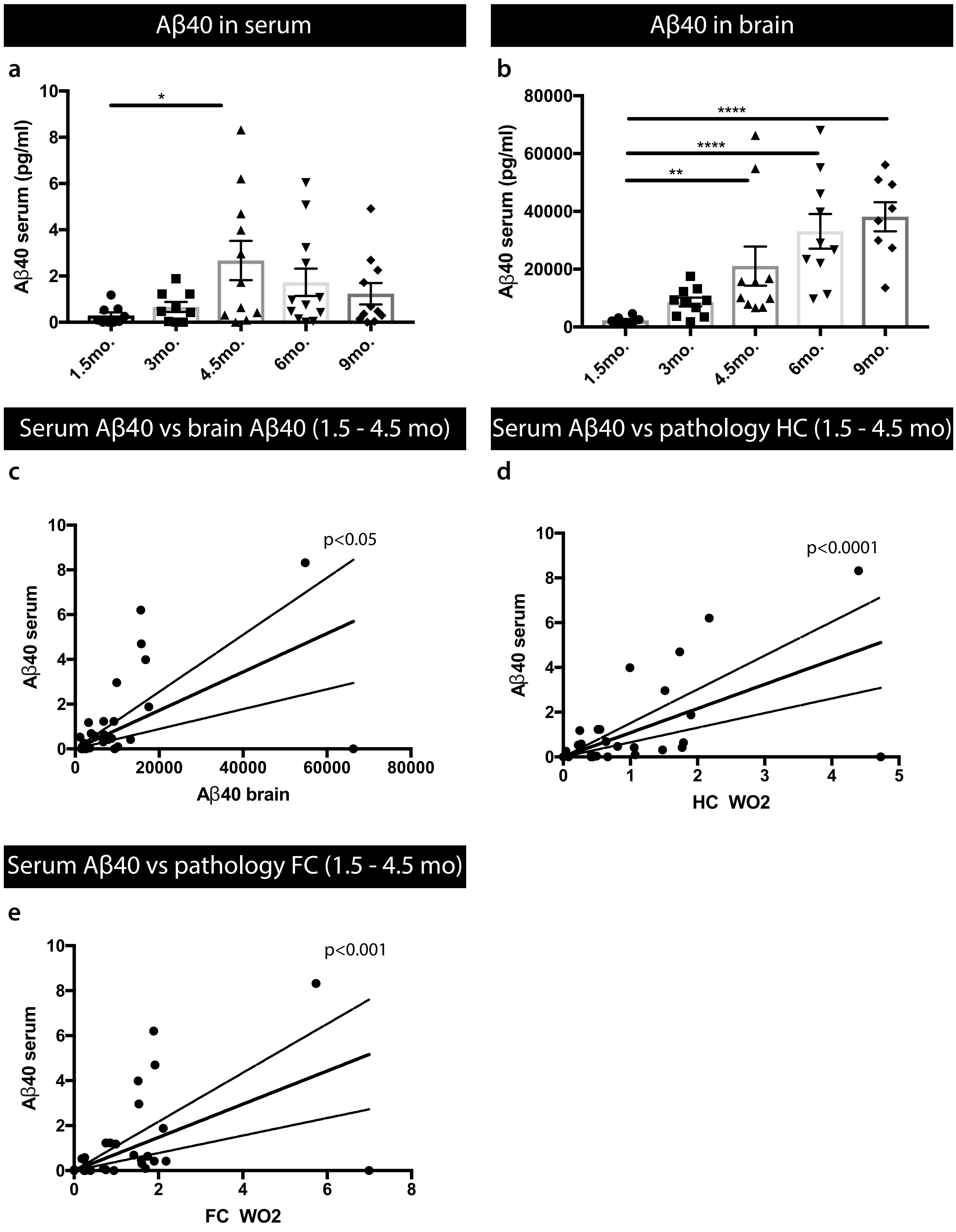

Figure S4. Relation of serum Aβ40 concentrations with amyloid pathology in different brain regions and Aβ40 levels in brain in 5xFAD mice

a) Aβ40 concentrations measured in serum of 1.5 mo, 3 mo, 4.5 mo, 6 mo and 9 mo old 5xFAD mice. Quantitative analysis reveals significantly higher concentrations in serum of 4.5 months 5xFAD mice compared to 1.5 months old mice. Kruskal–Wallis test with Dunn's multiple comparison (non-normally distributed). Data are presented as mean ± SEM; *$p < 0.05$; **$p < 0.01$; ***$p < 0.001$; ****$p < 0.0001$ (1.5, 3, 4.5, 6mo, 9mo: n=9, 9, 11, 12, 11) **b)** Brain Aβ40 concentrations were measured in total brain extracts using electrochemiluminescence detecting Aβ40 in the different age groups of 5xFAD mice. Quantitative analysis revealed a significant increase in 4.5 mo, 6 mo and 9 mo old mice compared to 1.5 months old mice. Kruskal–Wallis test with Dunn's multiple comparison (non-normally distributed) (1.5, 3, 4.5, 6mo, 9mo: n=8, 10, 10, 10, 8). Data are presented as means ± SEM; *$p < 0.05$; **$p < 0.01$; ***$p < 0.001$; ****$p < 0.0001$. **c, d, e)** Spearman´s correlation analysis between serum Aβ40 levels and brain Aβ40 levels (rs = 0.489, $p < 0.05$, n=26), and between serum Aβ40 levels and amyloid pathology (WO2) in HC (rs = 0.4759, $p < 0.05$, n=28) and FC (rs = 0.464, $p < 0.05$, n=28) at 1.5, 3 and 4.5 months old in 5XFAD mice. Linear regression analysis demonstrated a positive slope significantly different from 0 (for brain Aβ40 p<0,05 **(c)**, WO2 HC p<0,0001 **(d)** and WO2 FC p< 0,001**(e))**.

Figure S4

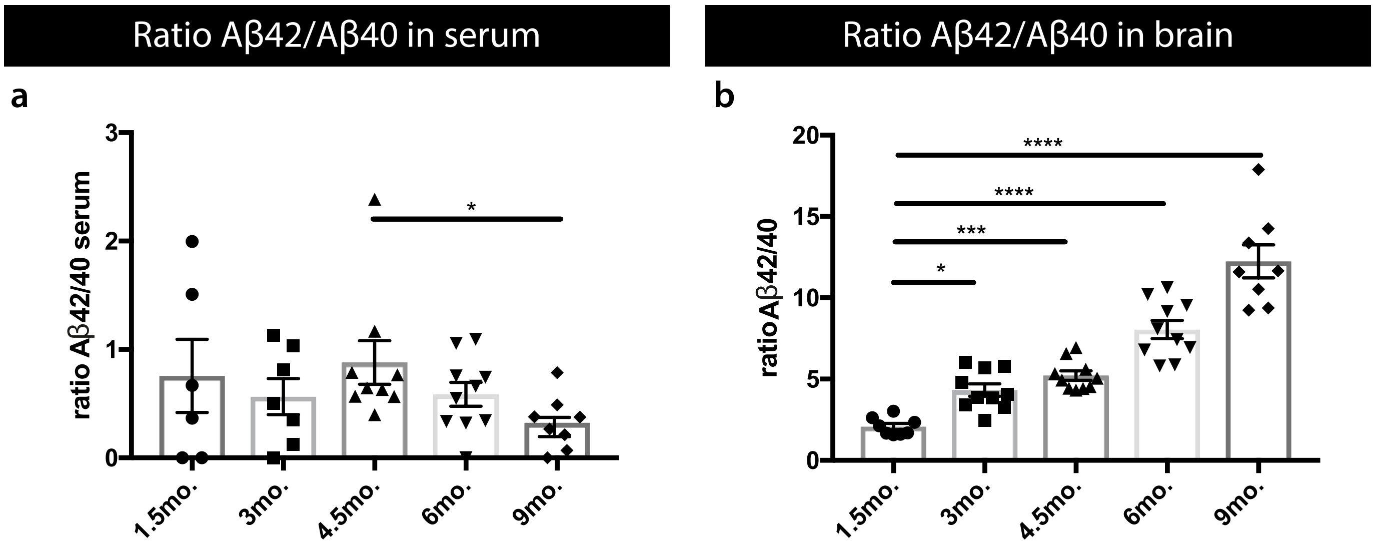

Figure S5. Serum Aβ42/40 ratio and Aβ42/40 ratio in brain in 5xFAD mice

a) Aβ42/Aβ40 ratios measured in serum of 1.5 mo, 3 mo, 4.5 mo, 6 mo and 9 mo old 5xFAD mice. Quantitative analysis reveals significantly lower Aβ42/Aβ40 ratios in serum of 9 months old 5xFAD mice compared to 4.5 months old mice. This indicates a decreased Aβ42/40 ratio in the presence of amyloid pathology. Kruskal–Wallis test with Dunn's multiple comparison (non-normally distributed). Data are presented as mean ± SEM; *$p < 0.05$; **$p < 0.01$; ***$p < 0.001$; ****$p < 0.0001$ (1.5, 3, 4.5, 6mo, 9mo: n=6, 7, 9, 10, 8), outliers identified using ROUT 0,1%, and missing values due to division by 0-value for Aβ40.

b) Brain Aβ42/Aβ40 ratios were measured in total brain extracts using electrochemiluminescence detecting Aβ40 in the different age groups of 5xFAD mice. Quantitative analysis revealed a significant increase in Aβ42/Aβ40 ratio 4.5 mo, 6 mo and 9 mo old mice compared to 1.5 months old mice. One-way ANOVA with Dunnett's multiple comparison (normally distributed) (1.5, 3, 4.5, 6mo, 9mo: n=8, 10, 10, 10, 8). Data are presented as means ± SEM; *$p < 0.05$; **$p < 0.01$; ***$p < 0.001$; ****$p < 0.0001$.

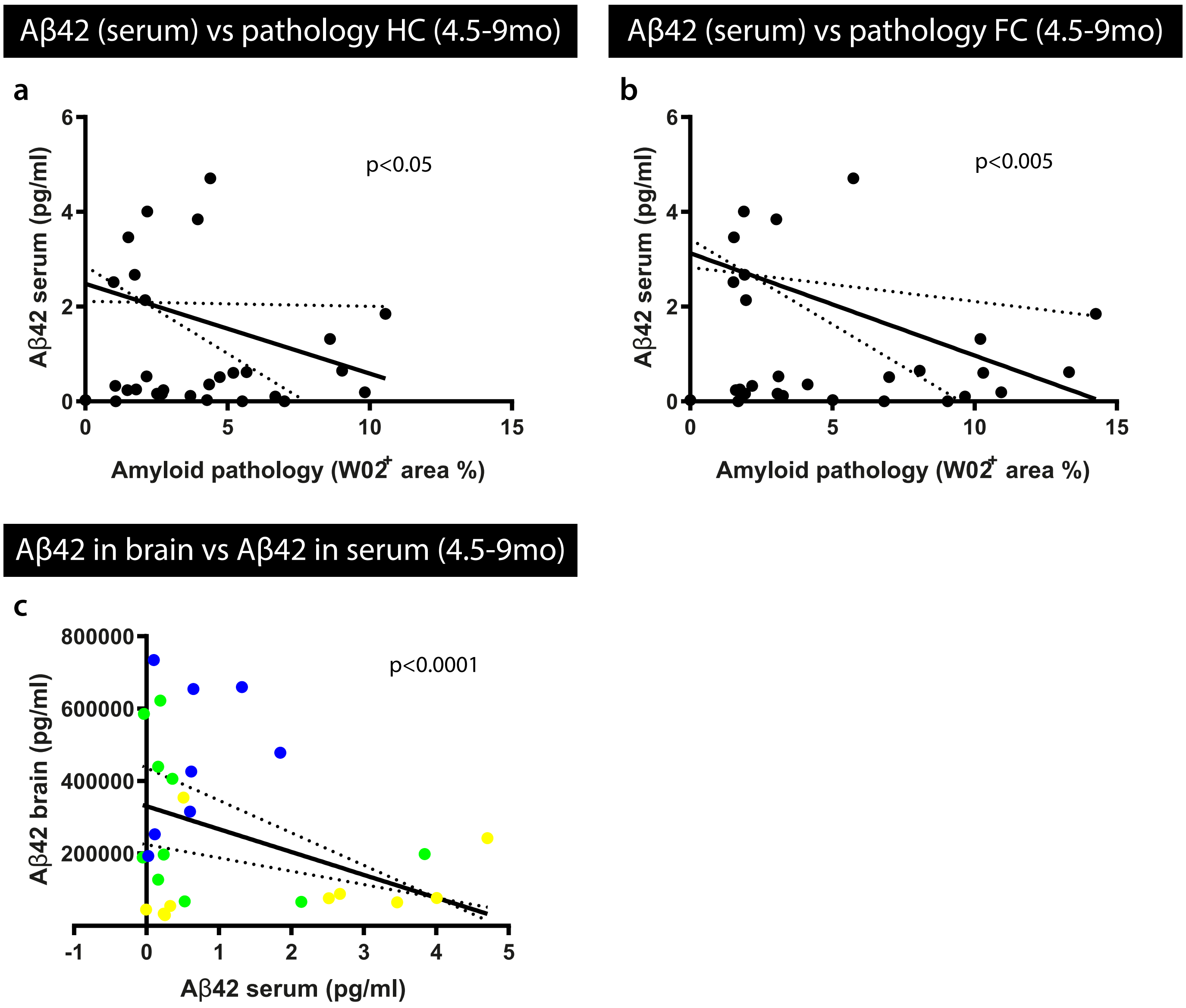

Figure S6. Relationship between pathology and serum Aβ42 levels in 5xFAD in the pathological phase.

a,b) Linear regression analysis between Aβ42 serum levels and pathology (WO2 positive area %) in **(a)** HC ($p < 0.05$, n=28) and **(b)** FrCx ($p < 0.005$, n=28) at 4.5, 6 and 9 months old in 5xFAD. **(c)** Linear regression analysis between Aβ42 levels in total brain homogenates and serum at 4.5 (yellow), 6 (green) and 9 (blue) months old in 5XFAD mice ($p < 0.0001$, n=28).

Figure S6

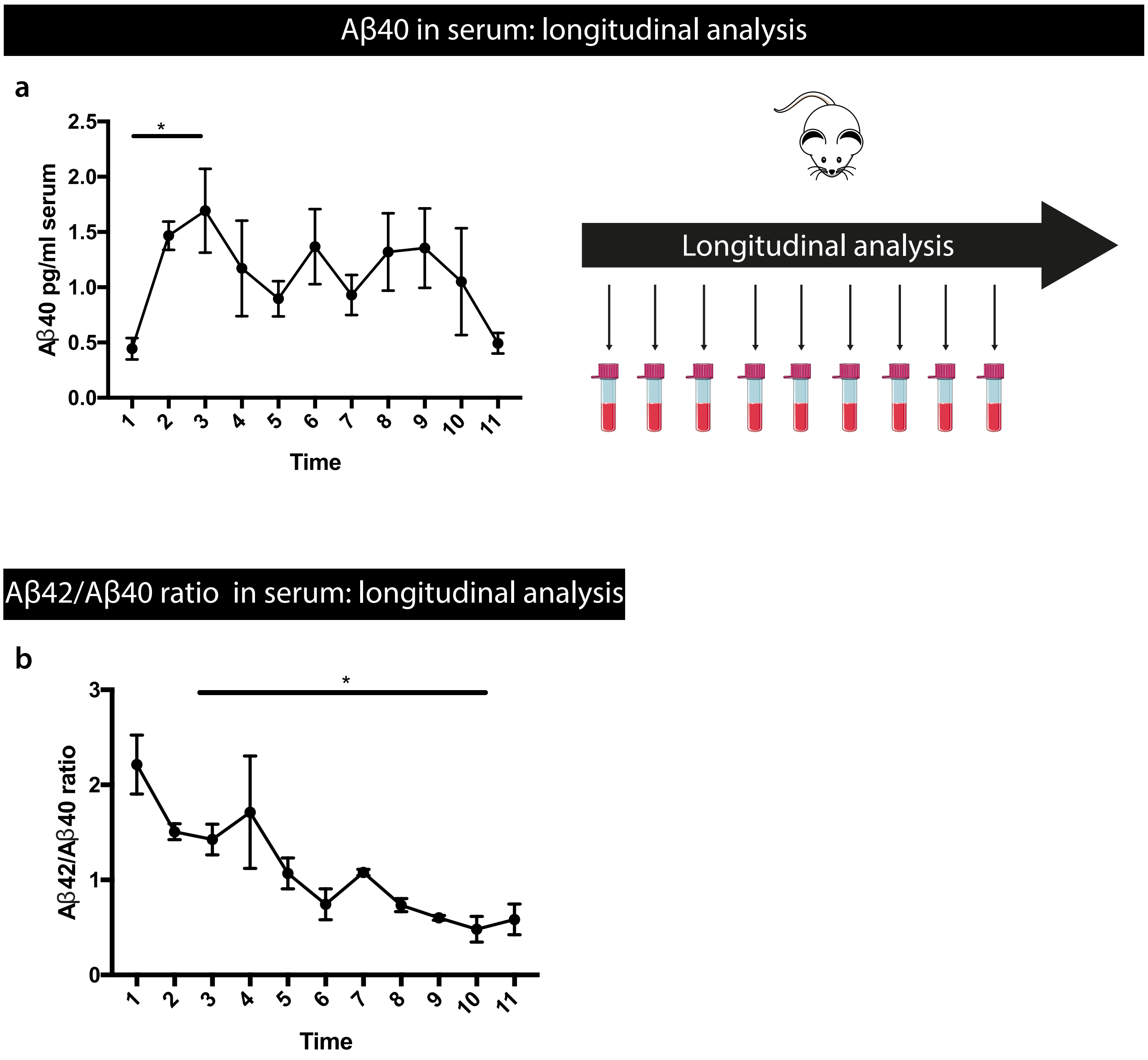

Figure S7 Longitudinal dynamics of serum Aβ in 5xFAD mice

a) Quantitative analysis of Aβ40 concentrations using electrochemiluminescence assay, of longitudinal serum samples, reveals an initial significant increase in Aβ40 serum concentrations, in the pre-pathological stage (11 time-points were collected starting from 2,5 months onwards). **b)** Calculated Aβ42/40 ratio of longitudinal serum samples reveals a significant decrease associated with the pathological stage. Repeated measures one-way ANOVA with Dunnett's multiple comparison (n=5 mice). Missing samples (n=6 out of 55) were filled in using the mean of values. Data are presented as mean ± SEM; *$p < 0.05$; **$p < 0.01$; ***$p < 0.001$; ****$p < 0.0001$.

Figure S7

	non-AD <50	non-AD >60	AD
n	16	13	6
age (years)	38,5 +/- 2,2	69,0 +/- 1,20	70,5 +/- 1,21
gender	9M/7F	9M/4F	4M/2F
CSF Aβ42	232,9 +/- 36,8 pg/ml	342,15 +/- 35,25 pg/ml	201 +/- 24,3 pg/ml
CSF Aβ40	7603,1 +/- 820,9 pg/ml	7676 +/- 719 pg/ml	8420 +/- 1196 pg/ml
CSF ratio Aβ42/Aβ40	0,032 +/- 0,003	0,0471 +/- 0,0036	0,0246 +/- 0,0014
CSF Tau	86,00 +/- 11,0 pg/ml	126,19 +/- 12, 077 pg/ml	327,78 +/- 70,69 pg/ml
CSF P-Tau	30,04 +/- 2,44 pg/ml	31,45 +/- 2,33pg/ml	55,74 +/- 8,94 pg/ml

Figure S8 Table of demographics of AD patients
Overview table shows demographics and biochemical data of human subjects.

Figure S8

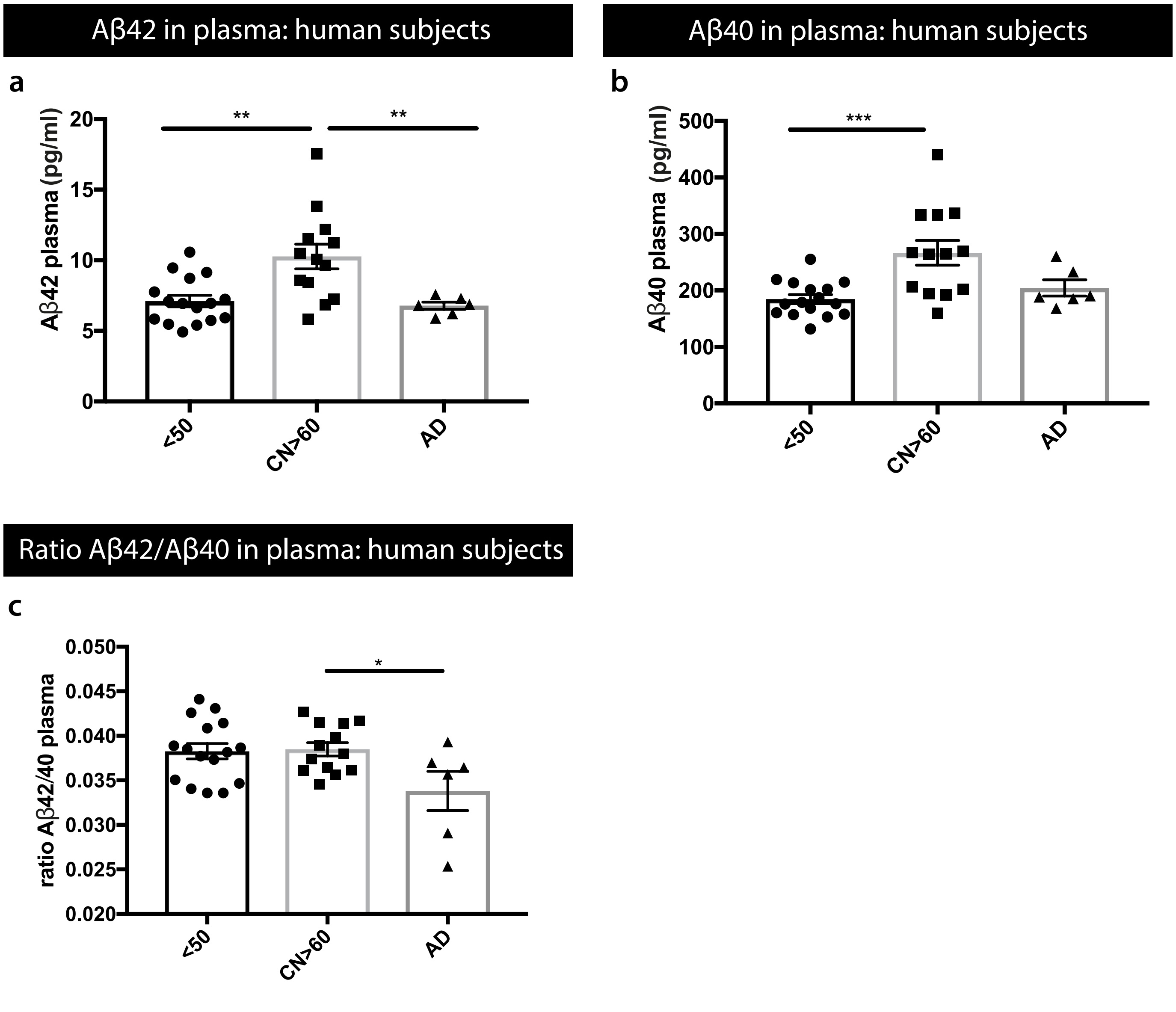

Figure S9. Aβ40 concentrations in plasma of human subjects

Measurements of Aβ42 **(a)** (retaken from Fig 9), Aβ40 **(b)** and ratio of Aβ42/Aβ40 **(c)** concentrations in plasma of human subjects, measured using SIMOA. Quantitative analysis reveals a significant higher Aβ42 plasma concentration and higher Aβ40 plasma concentration in >60 years controls, compared to <50years controls. Plasma Aβ42 and plasma Aβ42/Aβ40 is also significantly higher in >60 years controls compared to age-matched AD patients, while plasma Aβ40 is not significantly different. One-way ANOVA with Dunnett's multiple comparison test (normally distributed) (controls <50yrs, n=16; controls >60 years, n=13; AD patients n=6). Data are presented as mean ± SEM; *$p < 0.05$; **$p < 0.01$; ***$p < 0.001$; ****$p < 0.0001$.

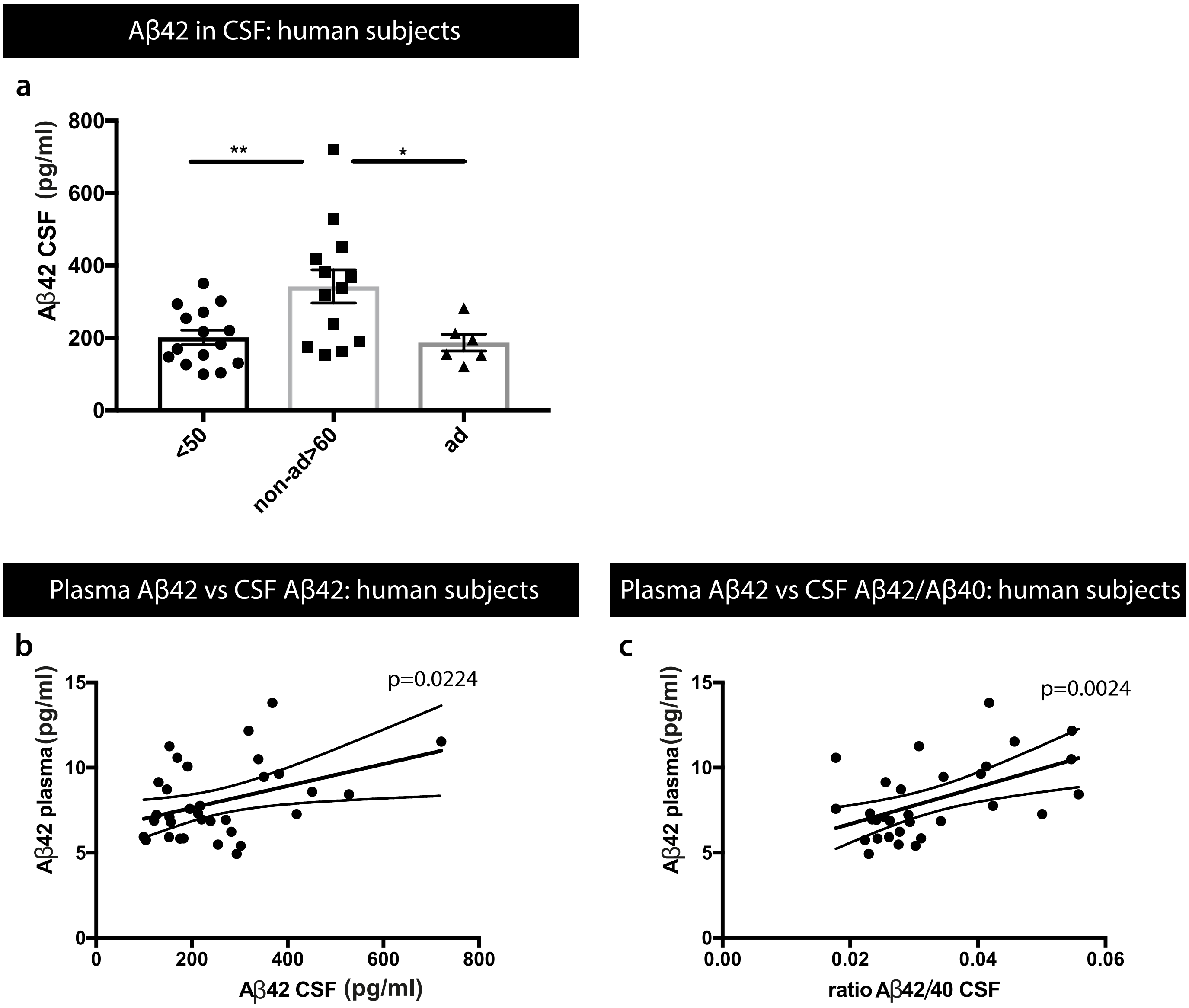

Figure S10. Aβ42 concentrations in CSF of human subjects and correlation of plasma Aβ42 with CSF Aβ42 and with CSF Aβ42/40 ratio in human subjects

a) Measurements of Aβ42 concentrations in CSF of human subjects, measured using SIMOA. Quantitative analysis reveals a significant higher Aβ42 CSF concentration in >60 years controls, compared to age matched AD patients, as well as compared to <50years controls. One-way ANOVA with Dunnett's multiple comparison test (normally distributed) (controls <50yrs, n=16; controls >60 years, n=13; AD patients n=6). 1 outlier identified using ROUT 0,1% in group <50yrs. Data are presented as mean ± SEM; *p < 0.05; **p < 0.01; ***p < 0.001; ****p < 0.0001. **b, c)** Pearson's correlation analysis between plasma Aβ42 levels and CSF Aβ42 levels (r = 0.3964, p= 0.0224) **(b)**, and between plasma Aβ42 levels and ratio Aβ42/ Aβ40 (r = 0.5248, p = 0.0024)(c). Linear regression analysis also demonstrated a positive slope significantly different from 0 (for CSF Aβ42 p<0,0001, ratio Aβ42/ Aβ40 p< 0,0001).

Figure S10

Blood-based biomarker analysis in preclinical models and human subjects

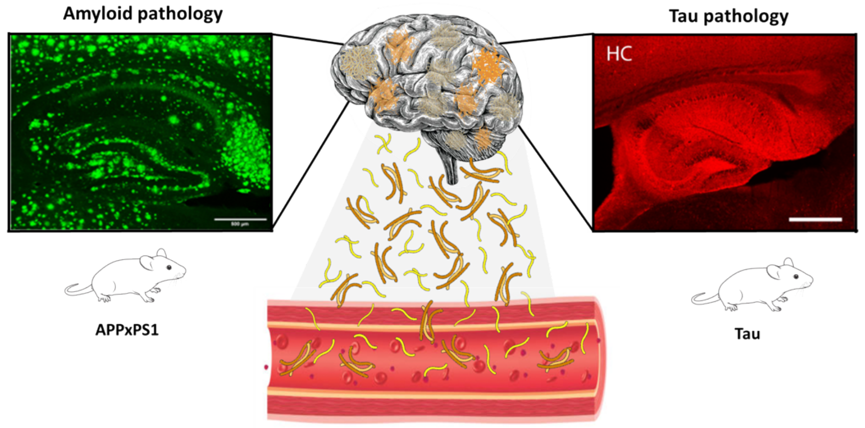

Combined blood based biomarker analysis in preclinical models and human subjects enables innovative insights due to well-defined predictable spatio-temporal pathology development in preclinical models and subsequent validation in small human cohorts

Early increase in blood-based Aβ42 in the earliest pre-pathological phase, before progressive amyloid pathology develops, correlates with Aβ42 in brain

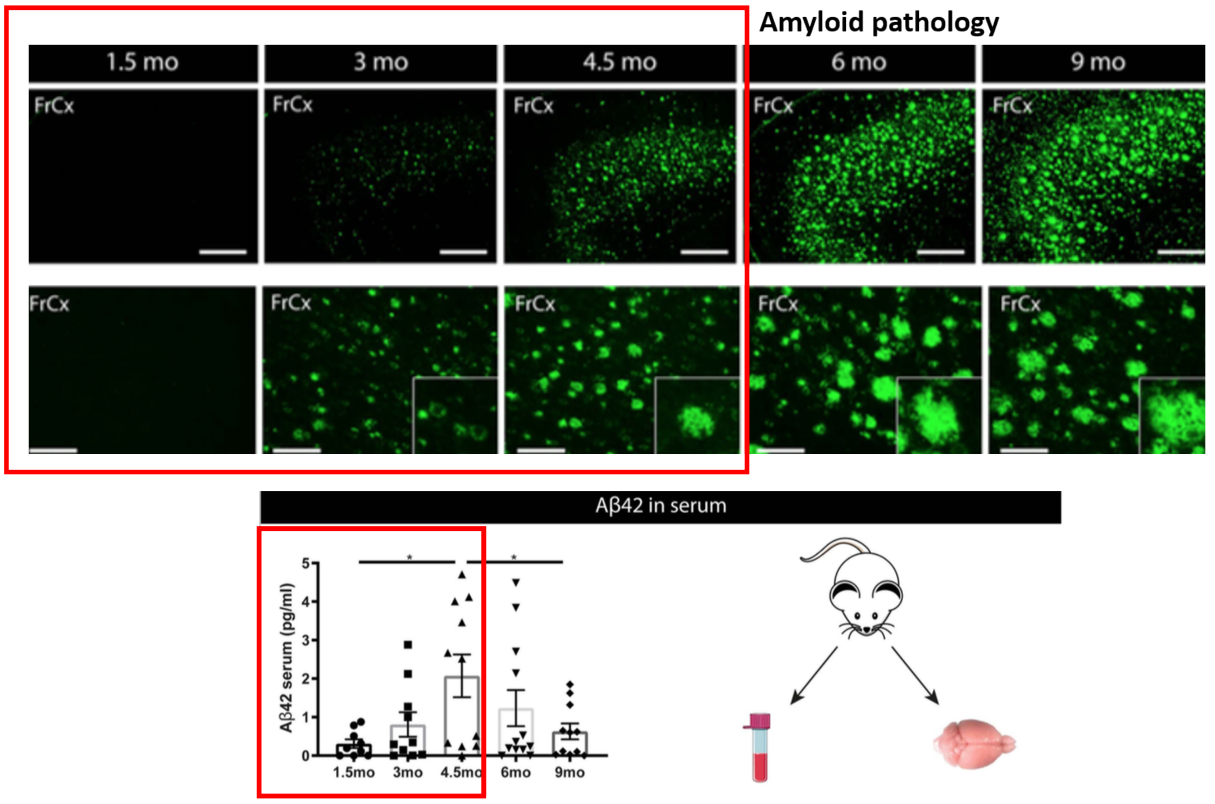

Towards prevention in the earliest pre-pathological phase: Prevention of Aβ42 accumulation before plaque deposition

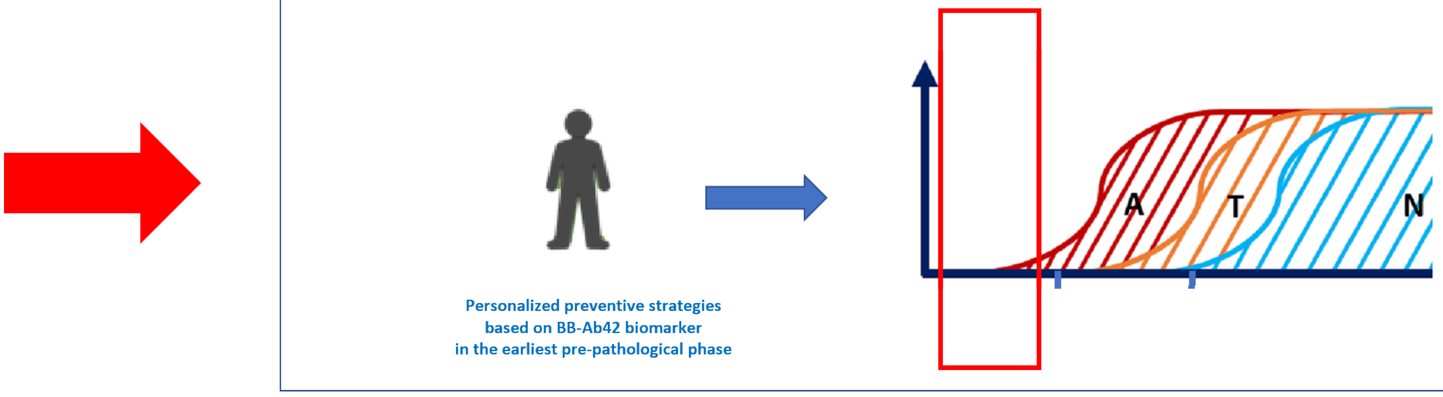

Personalized preventive strategies based on BB-Ab42 biomarker in the earliest pre-pathological phase

Overview Figure
